# Prevention of Influenza Virus-Induced Immunopathology by TGF-β Produced during Allergic Asthma

**DOI:** 10.1371/journal.ppat.1005180

**Published:** 2015-09-25

**Authors:** Yoichi Furuya, Andrea K. M. Furuya, Sean Roberts, Alan M. Sanfilippo, Sharon L. Salmon, Dennis W. Metzger

**Affiliations:** Center for Immunology and Microbial Disease, Albany Medical College, Albany, New York, United States of America; University of Iowa, UNITED STATES

## Abstract

Asthma is believed to be a risk factor for influenza infection, however little experimental evidence exists to directly demonstrate the impact of asthma on susceptibility to influenza infection. Using a mouse model, we now report that asthmatic mice are actually significantly more resistant to a lethal influenza virus challenge. Notably, the observed increased resistance was not attributable to enhanced viral clearance, but instead, was due to reduced lung inflammation. Asthmatic mice exhibited a significantly reduced cytokine storm, as well as reduced total protein levels and cytotoxicity in the airways, indicators of decreased tissue injury. Further, asthmatic mice had significantly increased levels of TGF-β1 and the heightened resistance of asthmatic mice was abrogated in the absence of TGF-β receptor II. We conclude that a transient increase in TGF-β expression following acute asthma can induce protection against influenza-induced immunopathology.

## Author Summary

Influenza and asthma represent the two major lung diseases in humans. While most studies have focused on exacerbation of asthma symptoms by influenza virus infection, the effects of asthma on susceptibility to influenza virus infections has been far less studied. Using a novel mouse model of asthma and influenza infection, we show that asthmatic mice are highly resistant to primary challenge with the 2009 influenza pandemic strain (CA04) compared to non-asthmatic mice. The increased resistance of asthmatic mice is not due to the enhanced T or B cell immunity but rather, to a strong anti-inflammatory TGF-beta response triggered by asthma. This study is the first to provide a mechanistic explanation for asthma-mediated protection during the 2009 influenza pandemic.

## Introduction

Influenza A virus is a respiratory pathogen that continues to circulate in humans, causing significant mortality and morbidity. Although antiviral drugs are available, the pandemic threat posed by influenza A viruses is expected to continue due to the lack of an effective long-term influenza vaccine. Despite a major influenza pandemic almost 100 years ago, we still lack a complete understanding of the genetic or physiological risk factors associated with influenza infections.

Asthma has been considered for many years to be a major risk factor for severe influenza infections. For this reason the Advisory Committee on Immunization Practices recommends that individuals who have chronic pulmonary disorders, including asthma, be prioritized for vaccination in the event of limited vaccine supply [1]. Asthma is a chronic lung disease characterized by progressively deteriorating lung function. Allergen exposure can induce asthma attacks that are characterized by repetitive cough and wheezing due to airway hyper-responsiveness and airway narrowing. Many allergens can exacerbate asthmatic symptoms including viruses such as human rhinovirus, respiratory syncytial virus and influenza viruses, which together account for approximately 75% of asthma attacks [2–5]. Thus, the ability of respiratory viruses to provoke asthmatic symptoms is a well-known phenomenon. Furthermore there is a strong association between respiratory viral infections in childhood and later onset of asthma development [6–9].

Survival following influenza virus infection is determined by host resistance, *i*.*e*., viral clearance by anti-viral immunity, and tolerance to the damage caused by the virus and host immunity [10]. More specifically, uncontrolled innate immune responses triggered by the virus can result in a fatal outcome from influenza virus infection in both humans [11,12] and animal models [13]. Indeed, virulent strains of influenza A viruses, such as avian H5N1 and Spanish 1918 H1N1, are known for their ability to elicit an excessive inflammatory response or a so-called “cytokine storm” [14]. Because host immune responses can play a pathogenic role during influenza infection, immune suppressive drugs, such as corticosteroids, have been traditionally used to treat severe cases of influenza infection. However, the efficacy of systemic corticosteroids remains controversial [15,16].

Transforming growth factor beta (TGF-β) is a known immunoregulatory cytokine. The importance of TGF-β in controlling inflammation has been demonstrated in TGF-β deficient mice, which exhibit spontaneous systemic inflammation resulting in postnatal lethality [17,18]. Respiratory viral infections can trigger secretion of TGF-β which plays an important role in attenuating pulmonary inflammation and thereby prolonging survival of the host [19,20]. Specifically, TGF-β promotes differentiation of Tregs which in turn produce additional TGF-β [21] and suppress effector functions of both innate and adaptive immune cells during influenza infections [22,23]. In addition, TGF-β is involved in tissue repair and re-modeling of the respiratory tract through stimulation of matrix protein production, epithelial proliferation and differentiation [24,25]. However, overproduction of TGF-β can be detrimental and has been implicated in development of fibrosis and thickening of airway walls in asthmatics [26].

Given that the prevalence of asthma has increased at an alarming rate, affecting 235 million people in 2011, the impact of asthma on host mucosal immunity warrants detailed investigation. In the present study, we utilized a mouse co-morbidity model of primary influenza infection and allergic airway inflammation to investigate the impact of asthma on host susceptibility to the 2009 pandemic H1N1 A/California/4/2009 (CA04) virus. To our surprise, we found that asthma transiently enhanced survival against primary influenza infection. This increased survival was mediated by high expression of the anti-inflammatory cytokine, TGF-β1. We further found that TGF-β1 mediated increased survival by preventing tissue injury during influenza infection and not by increasing viral clearance.

## Results

### OVA sensitized and challenged mice exhibit a typical asthmatic phenotype

**Fig 1:**
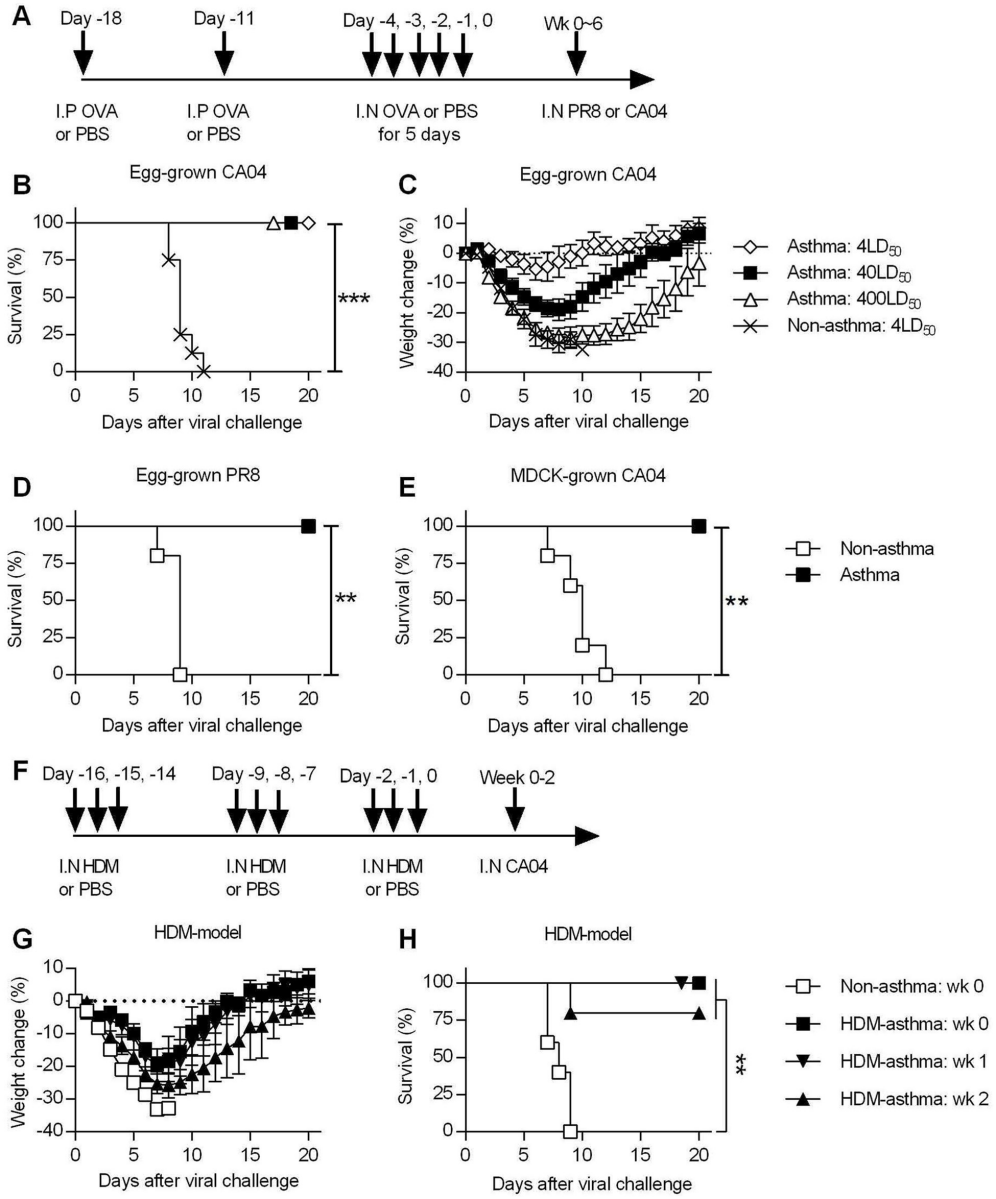
Asthmatic mice are highly resistant to influenza A virus challenge. (**A**) The general experimental procedure used in this study. Mice were first sensitized to OVA by two i.p. injections, followed by i.n. OVA treatment for 5 consecutive days to induce asthma. Control non-asthmatic mice received PBS only. Asthmatic and non-asthmatic mice were challenged i.n. with PR8 or CA04 influenza A virus at different time points after the last OVA treatment. (**B** and **C**) At day 7 after the last PBS or OVA treatment, asthmatic and non-asthmatic mice were infected with either 4, 40 or 400LD_50_ of CA04 influenza virus (8 mice/group). Infected mice were monitored for survival (B) and weight loss (C). (**D** and **E**) Groups of non-asthmatic and asthmatic mice were challenged with either egg-grown PR8 strain (2x10^3^ PFU) (D) or MDCK grown CA04 virus (6x10^4^ TCID_50_) (E). Infected mice were monitored for 20 days for survival (5 mice/group). (**F**) A mouse model of HDM-induced asthma. (**G** and **H**) HDM-induced asthmatic and non-asthmatic mice were i.n. challenged with CA04 virus either 1, 2, or 3 weeks following HDM challenge. Weight loss (**G**) and survival (**H**) were monitored for 20 days (5 mice/group). *P<0.05; **P<0.01; ***P<0.001.

To assess the impact of ovalbumin (OVA)-induced allergic airway inflammation on susceptibility to primary influenza A virus infection, we developed a comorbidity mouse model of asthma and influenza (Fig 1A). Asthma was induced by first sensitizing BALB/c mice intraperitoneally (i.p.) with OVA in aluminum hydroxide and then challenging intranasally (i.n.) with soluble OVA. PBS-treated mice were used as a negative control and will be referred to as non-asthmatic mice onwards. Histological analysis showed that the lungs of asthmatic mice were severely inflamed with extensive inflammatory cell infiltration on days 1 and 7 (S1A Fig). Also, inoculation of aerosolized methacholine increased airway resistance (*R*_*N*_), tissue resistance (*G*), and tissue elastance (*H*) in asthmatic, but not in non-asthmatic mice (S1B–S1D Fig). In addition, Th2 pro-inflammatory cytokines, such as IL-4, IL-5, and IL-13, were highly expressed on day 1 after the last OVA treatment of asthmatic mice but declined to levels similar to those seen in non-asthmatic mice by day 7 (S2 Fig). These results show that the OVA sensitization and challenge protocol induced a typical asthmatic phenotype. We also employed a mouse model of house dust mite (HDM)-induced asthma (S3A Fig). Similar to OVA, HDM treatment elicited a strong cellular infiltration, serum IgE responses, and airway hyperresponsiveness (S3B–S3D Fig). These two models allowed us to investigate the susceptibility of asthmatic mice to influenza virus infection. Asthmatic mice were routinely infected on day 7 after the last allergen treatment, unless otherwise indicated.

### Asthmatic mice are highly resistant to influenza A virus infection

To assess the susceptibility of asthmatic mice to influenza A virus infection, non-asthmatic and asthmatic mice were challenged with different doses of egg-grown CA04 2009 pandemic strain virus. While all non-asthmatic succumbed to a 4LD_50_ viral challenge dose, all asthmatic mice infected with doses of up to 400LD_50_ surprisingly survived challenge (Fig 1B). While a dose of 4LD_50_ caused little morbidity, i.e., minimal weight loss, in asthmatic mice, higher doses did cause noticeable morbidity around day 7 p.i., yet full recovery was observed by day 20 p.i (Fig 1C). Thus, asthmatic mice withstood high doses of the 2009 pandemic strain, demonstrating their remarkable resistance to influenza infection. This resistance was not specific to the CA04 strain, as asthmatic mice also survived lethal infection with the H1N1 A/Puerto Rico/8/1934 (PR8) virus (Fig 1D). In addition, we infected mice with MDCK cell-grown virus to determine whether the resistance of asthmatic mice was specific to egg-grown viruses. Consistent with the above results, asthmatic mice exhibited a high level of resistance to lethal challenge with MDCK-grown CA04 virus (Fig 1E). This shows that asthmatic mice are resistant to influenza infection and that this resistance is not dependent on conditions of viral propagation. Consistent with the OVA model, HDM-asthmatic mice also exhibited minimal morbidity (Fig 1G and S3E Fig) and enhanced resistance to CA04 infection (Fig 1H).

Endotoxin is a common contaminant of OVA [27] and HDM [28] preparations; LPS i.n. inoculation has been shown to be protective against influenza infections [29]. However, control, non-sensitized mice treated with i.n. OVA were not protected, demonstrating that the increased resistance was not caused by endotoxin contamination in the OVA preparation (S4 Fig). Furthermore, i.n treatment with *Escherichia coli* 0111:B4.9 LPS using an equivalent amount of endotoxin detected in our OVA stock did not confer resistance in mice (S3 Fig) Thus, the observed protection in OVA-asthmatic mice is not due to the endotoxin contamination.

### Asthma does not impact mucosal anti-viral humoral immunity

**Fig 2:**
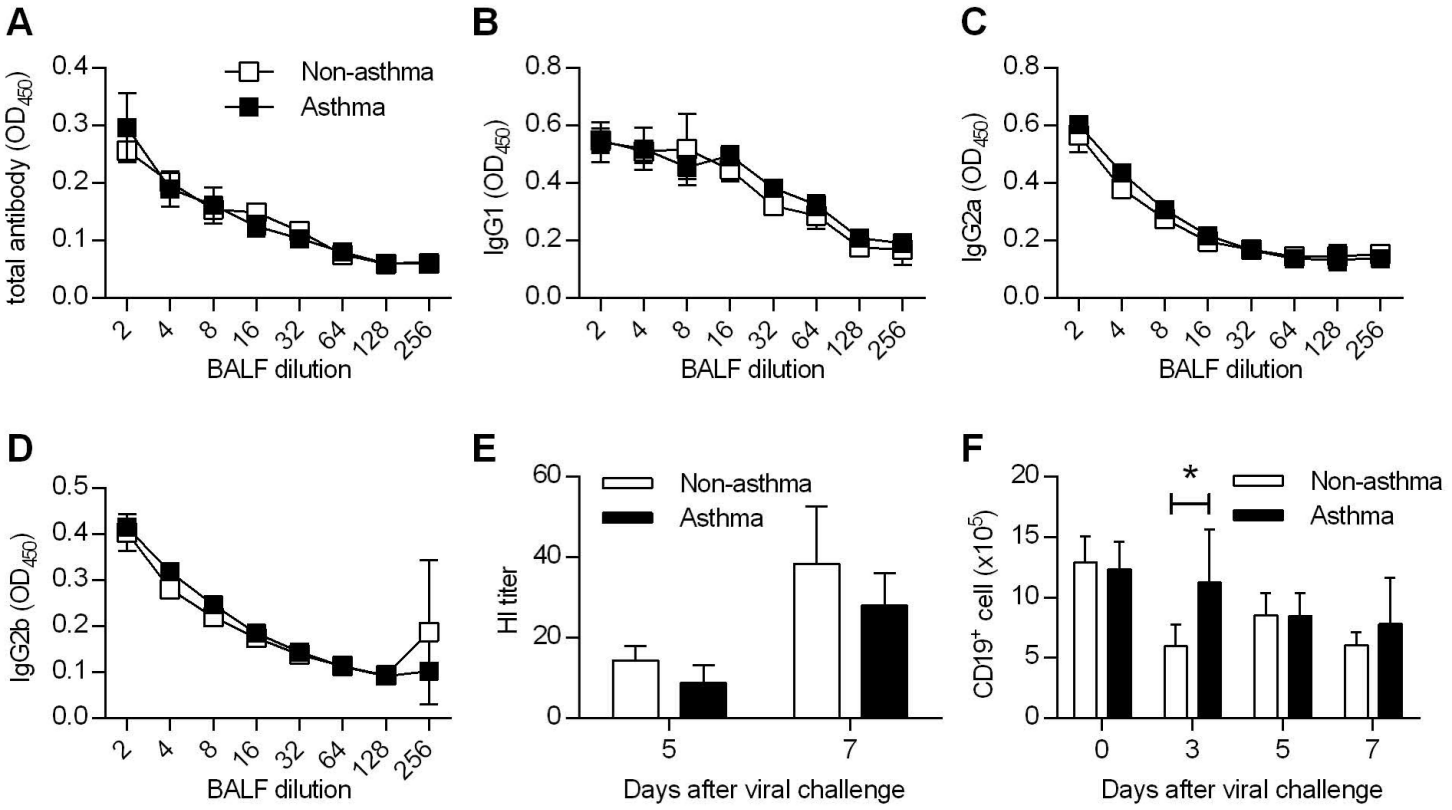
Comparable humoral immunity in non-asthmatic and asthmatic mice. (**A** to **D**) Non-asthmatic and asthmatic mice were infected with 4LD_50_ of CA04 virus. Seven days after primary challenge, BALF were tested for total (A), IgG1 (B), IgG2a (C), and IgG2b (D) antibody binding (4–5 mice/group). (**E**) Hemagglutination inhibition titers of BALF samples were determined (4–5 mice/group). (**F**) The numbers of CD19^+^ B cells were enumerated in the lung at various time points after CA04 virus infection (4 mice/group). *P<0.05.

To determine whether the increased resistance of asthmatic mice was due to increased adaptive immunity, we investigated the influence of asthma on virus-specific antibody responses. CA04-specific total antibody, IgG1, IgG2a, and IgG2b levels in BALF were comparable between non-asthmatic and asthmatic mice on day 7 p.i. (Fig 2A–2D). In addition, hemagglutination inhibition (HI) titers in non-asthmatic and asthmatic mice were similar on days 5 and 7 post-CA04 infection (Fig 2E). Analysis of CD19^+^ B cell expression showed that numbers of B cells in the lungs were also essentially comparable between the two groups (Fig 2F). Therefore, no changes in virus-specific humoral immune responses were observed that could account for the increased resistance observed following asthma.

### T cells are dispensable for enhanced protection to influenza virus infection in asthmatic mice

**Fig 3:**
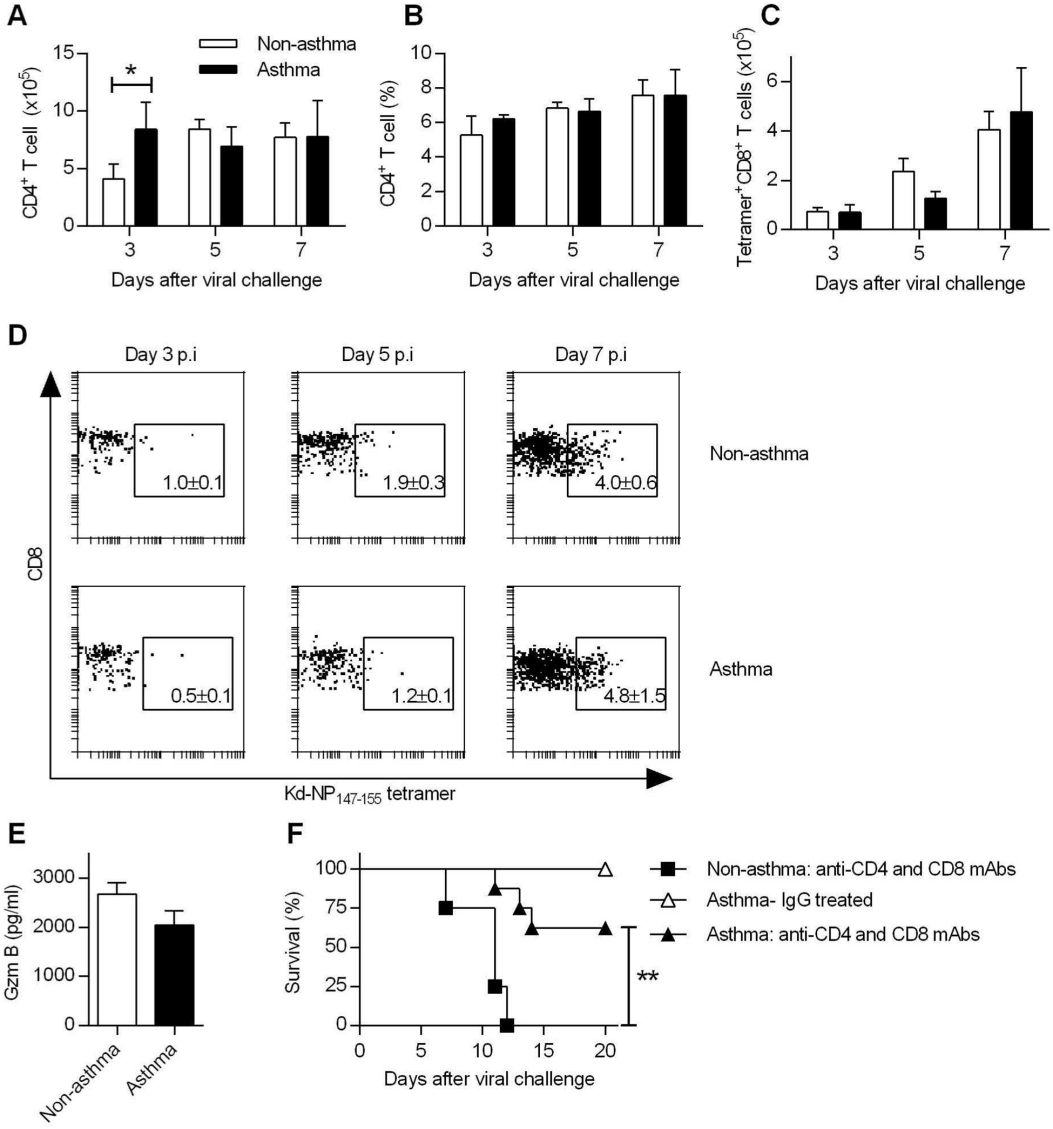
Increased resistance in asthmatic mice is T cell independent. (**A** to **D**) Groups of non-asthmatic and asthmatic mice were infected with CA04 virus on day 7 after the last OVA or PBS inoculation. Lungs were isolated on days 3, 5, and 7 post-infection and single cell suspensions were stained for CD4 (A and B), and nucleoprotein (NP)-specific CD8 (C) cells (4 mice/group). A representative dot plot for each group is shown with percentages of NP^+^CD8^+^ T cells in the lung (D). (**E**) BALF Gzm B levels on day 7 post-infection were quantified by ELISA (4–5 mice/group). (**F**) T cells were depleted by i.p. injection of GK1.5 and 53–6.72 mAb after the last PBS or OVA inoculation. Control mice were treated with rat IgG. Control and T cell-depleted mice were challenged with CA04 virus and monitored for survival (4–8 mice/group). *P<0.05, **P<0.01.

To examine the impact of asthma on mucosal T cell responses, lungs were harvested for flow cytometry at different time points to enumerate T cell recruitment. Absolute numbers of CD4^+^ T cells were higher in asthmatic mice relative to non-asthmatic mice on day 3 post-infection but were identical on days 5 and 7 (Fig 3A). Percentages of CD4^+^ T cells were also similar in non-asthmatic and asthmatic mice at all-time points tested (Fig 3B). Influenza A virus nucleoprotein (NP)-specific CD8^+^ T cell numbers were slightly higher in non-asthmatic mice on day 5 p.i. (Fig 3C). Similarly, the percentages of NP^+^CD8^+^ T cells were increased in non-asthmatic mice on days 3 and 5 but were equivalent on day 7 p.i. (Fig 3D). We further examined the amounts of the T cell effector molecule, granzyme (gzm) B in BALF. On day 7 p.i., which is the peak of the NP^+^CD8^+^ T cell response, levels of gzm B did not differ between asthmatic mice and non-asthmatic mice (Fig 3E). Overall, these results indicate that increased resistance in asthmatic mice was not attributed to altered T cell immunity.

In addition to virus-specific CD4^+^ T cells and CD8^+^ T cells responses, FoxP3^+^ CD4^+^ regulatory T cells (Tregs) and T helper 17 cells (Th17) are also induced during influenza infection. We observed differences in numbers of Tregs between non-asthmatic and asthmatic mice (S5A Fig). However, depletion of CD4^+^ or CD4^+^ and CD8^+^ T cells did not abrogate the resistance of asthmatic mice (Fig 3F and S5B and S5C Fig). In contrast, non-asthmatic mice depleted of CD4^+^ or CD4^+^ and CD8^+^ T cells had 100% mortality. Furthermore, mucosal L-17 responses were comparable between non-asthmatic and asthmatic mice (S5D Fig) and IL-17 receptor deficiency had no significant impact on survival of asthmatic mice (S5E Fig). Thus, CD4^+^/CD8^+^ T cells, Tregs or Th17 cells were not required for the enhanced resistance of asthmatic mice to CA04 infection.

### Innate immune responses are not required for the increased resistance of asthmatic mice to CA04 infection

**Fig 4:**
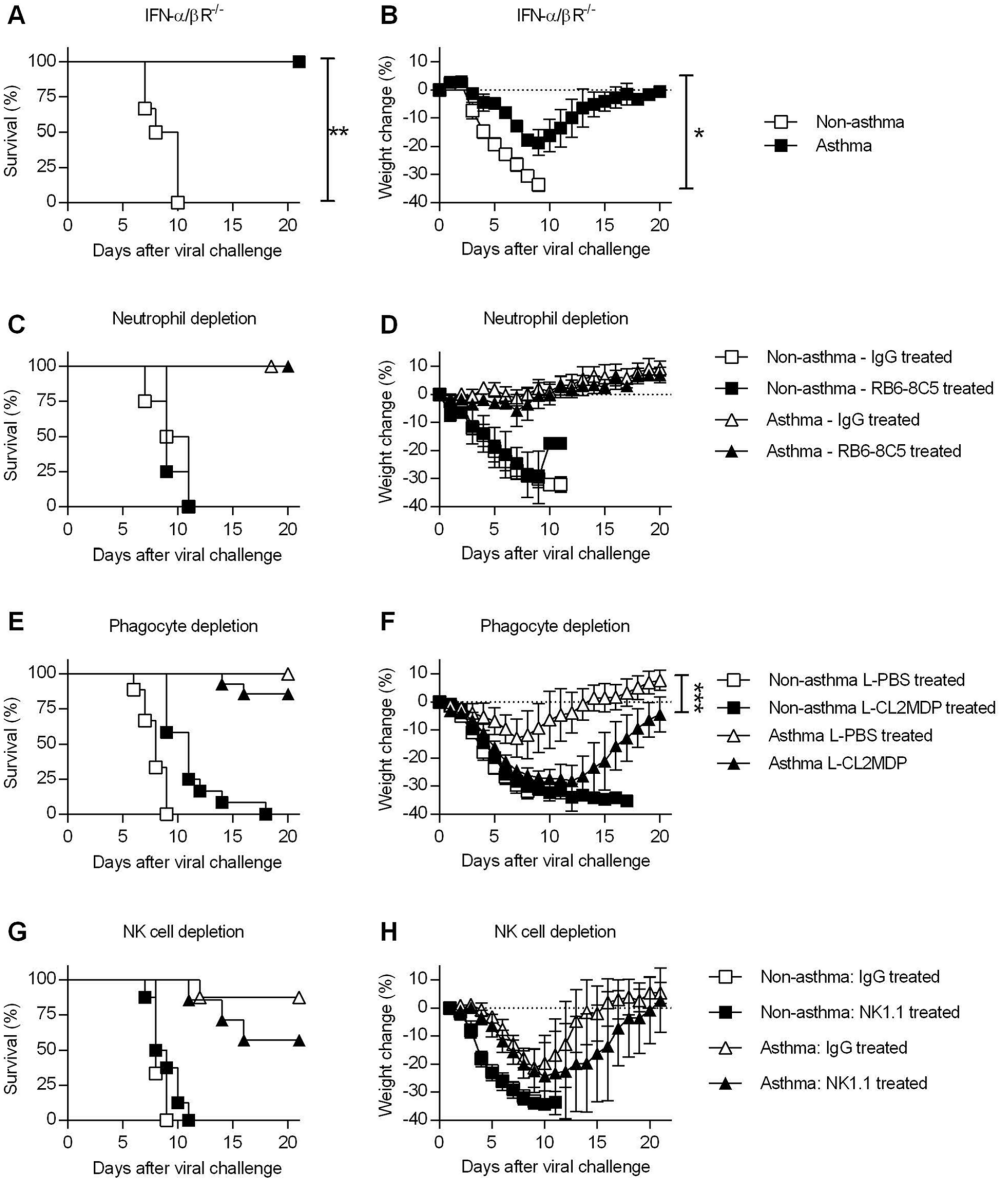
Innate immune responses are dispensable for the increased resistance of asthmatic mice. (**A** and **B**) OVA-induced allergic inflammation was induced in IFN-α/βR^-/-^ mice as described in the legend for Fig 1. Non-asthmatic and asthmatic IFN-α/βR^-/-^ mice were challenged with CA04 virus and were monitored for survival (A) and weight loss (B) (5–6 mice/group). (**C** to **H**) Non-asthmatic and asthmatic mice were depleted of various innate immune cells, infected, and monitored for survival. Mice were treated with RB6-8C5 rat mAb for neutrophil depletion (C and D) (4–6 mice/group), clodronate liposomes (L-CL2MDP) for phagocyte depletion (E and F) (9–14 mice/group), and NK1.1 rat mAb for NK cell depletion (G and H) (6–8 mice/group). Control mice were treated with rat IgG or PBS liposomes (L-PBS). Survival and weight loss were monitored for 20 days. *P<0.05, **P<0.01, ***P<0.001.

Since the resistance of asthmatic mice could not be attributed to the antigen-specific adaptive immunity, we next investigated the importance of innate immune responses. Because greater amounts of IFN-α, a potent antiviral cytokine, were found in asthmatic mice during the early stages of infection (S6A Fig), we used mice deficient in IFN-I receptor expression (IFN-α/βR^-/-^) to investigate the role of IFN-I in asthma-mediated resistance to influenza. Asthmatic IFN-α/βR^-/-^ mice survived a lethal challenge of CA04 virus while non-asthmatic mice succumbed to infection (Fig 4A). Consistent with the survival data, asthmatic IFN-α/βR^-/-^ mice had lower weight loss compared to non-asthmatic mice following CA04 infection (Fig 4B). Thus, absence of IFN-I signaling did not compromise the resistance of asthmatic mice.

We also performed a series of *in vivo* depletion experiments to investigate the contribution of various innate immune cells to asthma-induced resistance. Following i.n. challenge with OVA or PBS, asthmatic and non-asthmatic mice were treated with RB6-8C5 mAb, liposomal clodronate, or NK1.1 mAb to deplete neutrophils, macrophages, or NK cells, respectively. Control mice received normal rat IgG or liposomal PBS. After neutrophil depletion, there was no effect in asthmatic or non-asthmatic mice (Fig 4C and 4D). Phagocytic cell depletion did not significantly reduce the survival rate of asthmatic mice compared to liposomal PBS-treated asthmatic mice (Fig 4E) but did increase morbidity (Fig 4F). NK cell depletion had no significant effect on either survival or weight loss of non-asthmatic and asthmatic mice (Fig 4G and 4H). Thus, although altered recruitment of innate immune cells was observed in asthmatic mice (S6B–S6D Fig), individual innate immune cell types did not contribute significantly to the increased resistance of asthmatic mice.

### Enhanced survival is correlated with reduced immunopathology but not viral burden in asthmatic mice

**Fig 5:**
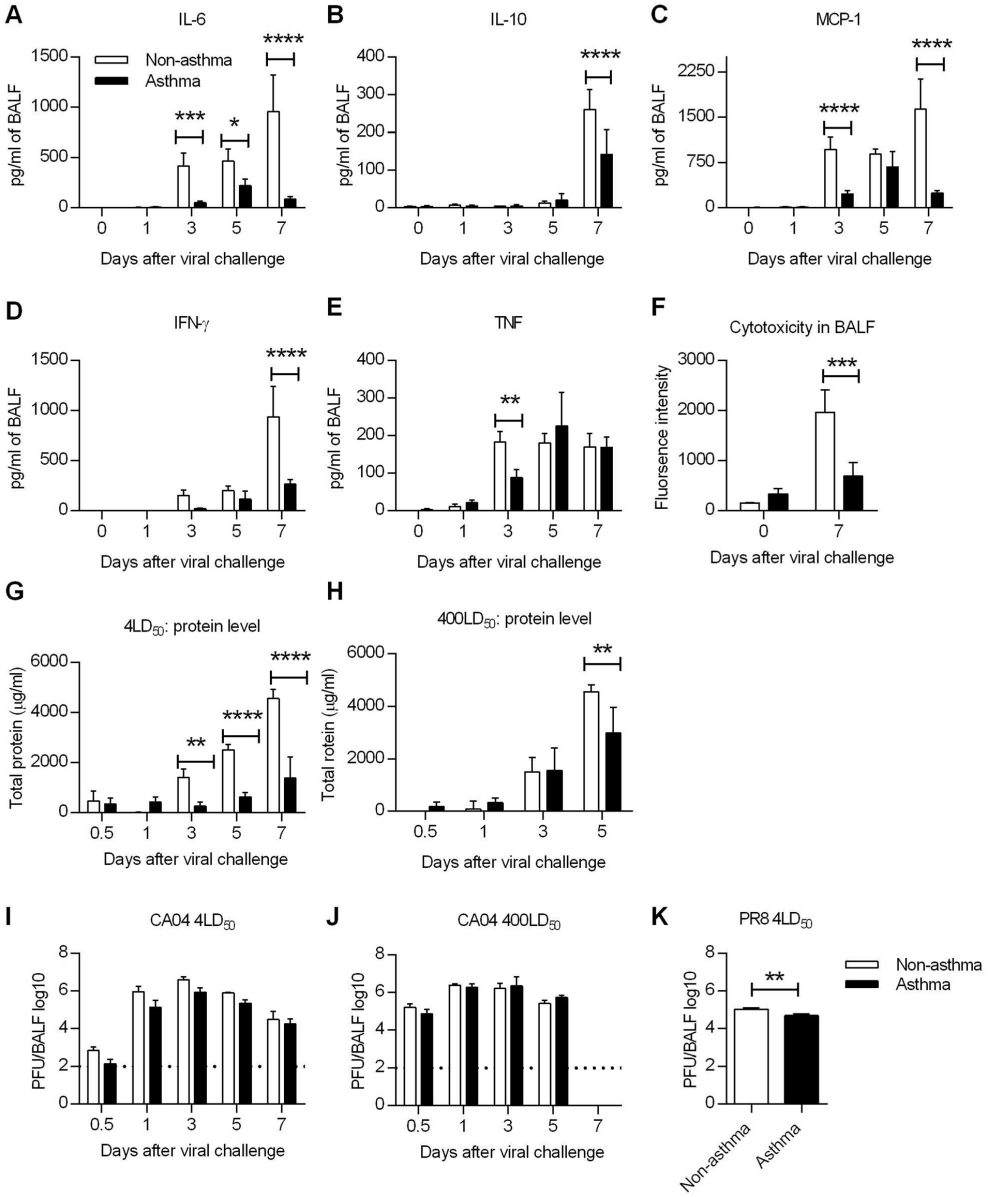
Influenza virus induced immunopathology is reduced in asthmatic mice. Non-asthmatic and asthmatic mice were infected i.n. with 4LD_50_ of the CA04 virus on day 7 after the last PBS or OVA treatment. The lungs were harvested and homogenized for cytokine quantification at various times following influenza infection. (**A** to **E**) Pulmonary cytokine levels were measured by cytometric bead array. (**F**) Cytotoxicity in BALF was determined by measuring glucose 6-phosphate. (**G** and **H**) Total protein levels in BALF were measured using the Pierce BCA Protein Kit following challenge with 4 LD_50_ (G) or 400LD_50_ (H) of CA04. (**I to K**) Viral burdens were determined in BALF after challenge with either 4 LD_50_ (I) or 400LD_50_ (J) of CA04 or 4LD_50_ of PR8 (K). Each bar indicates mean ± SD of 3–5 mice/group. *P<0.05, **P<0.01, ***P<0.001, ****P<0.0001.

The results presented above indicated that the increased resistance of asthmatic mice was not due to enhanced anti-viral immunity and suggested that virus-induced immunopathology might be downregulated in asthmatic mice, which could account for the observed increased survival rates. Although pro-inflammatory cytokines are integral components of an effective immune response against infection, excessive production can cause tissue damage and is associated with mortality [30]. Thus, we next tested whether the extent of the cytokine storm was decreased in asthmatic mice. Following a lethal infection with CA04 virus, protein levels of various bronchoalveolar lavage fluid (BALF) cytokines were quantified by cytometric bead array. We found that asthmatic mice had lower levels of IL-6, IL-10, MCP-1, IFN-γ, and TNF compared to non-asthmatic mice (Fig 5A–5E). Similar trends were observed using PR8 virus, with asthmatic mice producing significantly decreased amounts of cytokines (S7 Fig). The type 2 innate lymphoid cell-associated cytokines, amphiregulin, IL-5, and IL-13, were also downregulated in asthmatic mice (S8 Fig). Thus, enhanced survival of asthmatic mice correlates with reduced cytokine responses.

Since cytokine analysis showed decreased inflammatory responses in asthmatic mice, we next determined if tissue damage was also attenuated. To address this, BALF samples collected from uninfected and day 7-infected mice were tested for cytotoxicity and total protein levels. Cytotoxicity testing, which measures the extent of dying and damaged cells, revealed that cytotoxicity was significantly reduced in asthmatic mice on day 7 p.i. compared to that in non-asthmatic mice although baseline cytotoxicity was higher in asthmatic mice compared to non-asthmatic mice (Fig 5F). Total protein, another marker of lung injury and edema, was greater in asthmatic mice only at day 1 p.i.; levels were thereafter significantly less compared to those in non-asthmatic mice (Fig 5G). Even with a challenge dose of 400LD_50_, asthmatic mice had reduced amounts of protein in the BALF on day 5 p.i. compared to non-asthmatic mice (Fig 5H). Interestingly, influenza virus infection triggered reduced tissue damping and elastance in asthmatic mice, suggesting that the pulmonary function is less compromised in asthmatic mice (S9 Fig). Taken together, these results demonstrate that lung injury was significantly reduced in asthmatic mice as evaluated by cytokine production, cytotoxicity and protein levels in BALF.

To determine whether the reduced immunopathology of asthmatic was due to increased viral clearance, asthmatic and non-asthmatic mice were infected i.n. with either 4LD_50_ or 400LD_50_ of CA04 and BALF samples were collected for virus PFU enumeration. Viral replication was observed in both non-asthmatic and asthmatic mice. Although with a challenge dose of 4LD_50_, viral titers were approximately a half-log less in asthmatic mice on days 0.5, 1, 3 and 5 p.i., the differences were not statistically significant (Fig 5I and 5J). Similarly, after PR8 infection, day 7 viral titers were only slightly different between non-asthmatic and asthmatic (Fig 5K). These data show that the reduced susceptibility of asthmatic mice correlated with reduced immunopathology but not with viral burden. Consistent with the viral burden data, lung epithelial cell expression of sialic acid (SA) receptors (α-2,3 SA and α-2,6 SA), which meditate influenza viral entry, were unaltered in asthmatic mice (S10A and S10B Fig).

### Conditional deletion of TGF-β receptor II renders asthmatic mice susceptible to CA04

**Fig 6:**
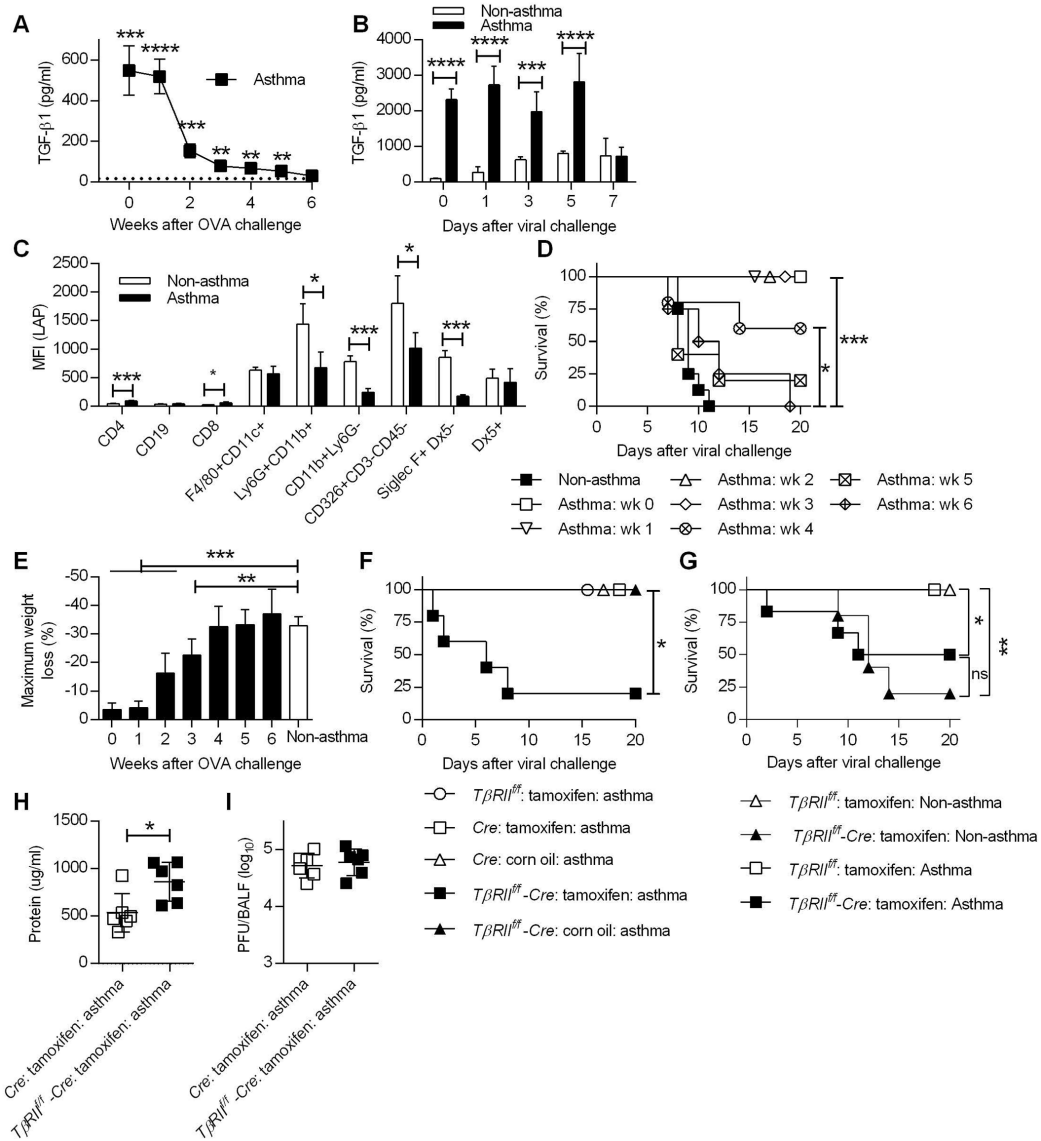
TGF-β mediates increased resistance in asthmatic mice. (**A**) TGF-β1 levels in BALF of noninfluenza-infected asthmatic mice (4 mice/group) were determined by ELISA. The dotted line represents background TGF-β1 levels in BALF of non-asthmatic mice. (**B**) TGF-β1 levels in BALF after CA04 infection were determined by Luminex (3–4 mice/group). (**C**) Cell surface expression of LAP was measured on day 7 after the last OVA inoculation (4 mice/group). (**D** and **E**) Asthmatic mice (5–8 mice/group) infected at different time points after the last OVA treatment were monitored for survival (D) and weight loss (E). (**F** and **G**) Survival of *TβRII* ^*f/f*^ *-Cre* and control mice were monitored for survival after infection of either 2x10^3^ PFU (F) or 50 PFU (G) of CA04 (4–8 mice/group). (**H** and **I**) Total protein levels (H) and viral burdens (I) in BALF were measured on day 3 post-infection (6 mice/group). *P<0.05, **P<0.01, ***P<0.001, ****P<0.0001.

One key cytokine that plays pro- and anti-inflammatory roles during asthma pathogenesis is TGF-β1 [26]. TGF-β1 initiates and regulates inflammatory responses as well as airway remodeling [31]. Given the potent immunoregulatory properties of TGF-β1, we determined whether TGF-β1 in asthmatic mice could account for reduced immunopathology following influenza infection. To examine this, TGF-β1 levels in BALF following i.n. OVA treatment were measured by ELISA. The peak of TGF-β1 expression in asthmatic mice was during weeks 0 and 1 post-OVA challenge (Fig 6A), and significantly declined by week 2. TGF-β1 levels continued to decrease during weeks 3, 4 and 5 but were still higher than the baseline levels detected in non-asthmatic mice. By week 6, the amounts of TGF-β1 in asthmatic mice had declined to background levels. We next quantified TGF-β1 protein levels following CA04 challenge. Consistent with the above findings, asthmatic mice had significantly higher levels of TGF-β1 on days 0, 1, 3, and 5 p.i. compared to non-asthmatic mice (Fig 6B). By day 7 p.i., TGF-β1 levels were comparable between non-asthmatic and asthmatic mice.

Because increased levels of TGF-β1 were detected in asthmatic mice, we next sought to identify the cells responsible for TGF-β1 production. It is widely accepted that TGF-β1 is constitutively produced in a biologically inactive pro-TGF-β1 form, which exists in complex with latency-associated protein (LAP) as a latent-TGF-β1 [32]. Therefore, staining of cells with anti-LAP mAb, which recognizes LAP, pro-TGF-β and latent-TGF-β, can be used to identify the cellular source of TGF-β. To examine this, we harvested lungs of non-asthmatic and asthmatic mice on day 7 post-OVA challenge for flow cytometry analysis and median fluorescence intensity (MFI) of LAP was quantitated for various cell types. The majority of LAP-expressing cells were innate immune cells (*e*.*g*., F4/80^+^CD11c^+^, Ly6G^+^CD11b^+^, CD11b^+^ Ly6G^-^, Siglec-F^+^DX5^-^ cells) and epithelial cells (CD326^+^CD3^-^CD45^-^ cells) (Fig 6C). LAP expression by these cell types was lower in asthmatic mice compared to non-asthmatic mice, suggesting ongoing secretion of TGF-β1, which correlates with lower cell surface expression as observed by others in a mouse model of asthma [33]. This is also in agreement with clinical data showing that cells recovered from asthmatic patients secrete greater amounts of TGF-β1 than cells from non-asthmatic patients [34,35]. The above findings led us to hypothesize that TGF-β1 suppresses immune-mediated tissue injury and thereby prevents influenza-induced mortality. To test this hypothesis, we first investigated if TGF-β1 production correlated with the window of resistance to influenza infection following asthma induction. At various time points after OVA treatment, asthmatic mice were i.n. infected with a lethal dose of CA04 virus, and survival and weight loss were monitored for 20 days. Survival analysis showed that the increased resistance of asthmatic mice to influenza declined over time (Fig 6D). Asthmatic mice that were infected on week 0, 1, 2, or 3 post-OVA treatment had a 100% survival rate. The survival rate decreased when asthmatic mice were challenged with influenza on week 4, however the survival rate of these mice was still significantly higher when compared to non-asthmatic mice. Finally, when influenza infection was performed on week 5 or 6 post-OVA treatment, survival rates of asthmatic and non-asthmatic mice were comparable. Consistent with the survival data, weight loss patterns increased when asthmatic mice were infected at later time points post-OVA challenge (Fig 6E). Overall, the reduced susceptibility of asthmatic mice was not permanent but declined over time and closely correlated with BALF TGF-β1 levels.

To directly determine the protective role of TGF-β1 against influenza infection, we utilized conditional TGF-β receptor II (TGF-βRII) deficient mice. Constitutive deficiency in TGF-β1 or TGF-βRII is embryonically lethal due to uncontrolled spontaneous inflammation [36,37]. To circumvent this issue, we created conditional knockout mice by crossing *TβRII* ^*f/f*^ mice with *Ubc-CreER* ^*T2*^ mice to generate tamoxifen-inducible TGF-βRII deficient mice *(TβRII* ^*f/f*^-*Cre)*. PCR analysis demonstrated tamoxifen-induced deletion of the floxed *TβRII* allele and quantitative evaluation by flow cytometry confirmed a significant reduction in *TβRII* expression (S11 Fig). Using this conditional knockout mouse, we showed that deletion of TGF-βRII in asthmatic mice resulted in decreased survival after CA04 virus infection while all of the wildtype asthmatic mice had 100% survival (Fig 6F). The protective role of TGF-β during influenza infection [19] complicates assessment of TGF-β as a mediator of increased resistance in asthmatic mice. To overcome this problem, we normalized infection to the CA04 virus LD_50_ for *TβRII* ^*f/f*^ deficient mice (Fig 6G). Infection of non-asthmatic *TβRII* ^*f/f*^ deficient mice with 50 PFU of CA04 virus resulted in 20% survival and asthma provided only marginally increased protection in these animals. Furthermore, asthmatic *TβRII* ^*f/f*^ deficient mice had increased total protein levels in BALF on day 3 post-viral challenge (Fig 6H), despite having viral burdens that were similar to wild-type asthmatic mice (Fig 6I). We conclude that deletion of *TβRII* abolished the resistance of asthmatic mice to influenza virus infection and that TGF-β mediated resistance is likely to be due to suppression of influenza virus-triggered immunopathology.

## Discussion

In the present study, asthmatic mice were found to be highly resistant to influenza A virus infection, including the 2009 influenza pandemic strain. It is particularly surprising that asthmatic mice were able to resist H1N1 CA04 virus infection at doses as high as 400LD_50_ which corresponds to 2 x 10^5^ viral PFU. This extraordinary resistance did not depend on the viral strain as asthmatic mice were also resistant to influenza PR8 virus. However, enhanced resistance was transient and lasted no longer than 4 weeks following acute asthma. Interestingly, no correlation was seen between increased survival and viral burden. This strongly suggests that the increased survival of asthmatic mice was not due to enhanced antiviral immunity but rather to increased tolerance to influenza infection-mediated tissue damage.

Influenza can kill the host either by direct pathology that is mediated by viral replication or by inducing a damaging inflammatory response. The absence of a correlation between viral burden and survival rate seen in asthmatic mice suggests that the induced protection was due to suppression of virus-induced immunopathology. This hypothesis was supported by reduced lung injury in asthmatic mice as determined by pulmonary cytokine expression, airway protein levels (indicative of severe edema), and cell death as measured by a cytotoxicity assay. These assays clearly indicated that asthmatic mice suffered less severe virus-mediated immunopathology. Consistent with our animal data, *ex vivo* study using primary human bronchial cells have shown that epithelial cells from asthmatic donors are also resistant to influenza virus-mediated pathology [38].

The two main cytokines associated with an anti-inflammatory state are IL-10 and TGF-β. In our co-morbidity mouse model of asthma and influenza, IL-10 was absent prior to CA04 infection, however, it was produced at later time points, *i*.*e*., day 7 p.i. Thus, it is reasonable to believe that IL-10 was expressed in response to tissue injury, with the lower levels observed in asthmatic mice correlating with reduced lung tissue pathology compared to non-asthmatic mice. In contrast, high levels of TGF-β1 were detected in asthmatic mice prior to infection. This is consistent with numerous human [39–42] and mouse studies [43], showing that TGF-β1 is transiently produced in large amounts during and after asthma exacerbations. Thus, we speculated that increased expression of TGF-β1 could mediate the increased survival of asthmatic mice through suppression of harmful immune responses. Indeed, the kinetics of TGF-β1 expression in BALF correlated strongly with longevity of resistance in asthmatic mice and its continuing increased production during influenza virus infection. Most importantly, a strong link between TGF-β1 and reduced susceptibility of asthmatic mice to influenza virus infection was demonstrated using conditional TGF-βRII knockout mice in which deletion of the *TβRII* allele completely abrogated the resistance of asthmatic mice. Consistent with our observations, others have reported that *in vivo* neutralization of TGF-β increases susceptibility to infection to both H5N1 virus and the 2009 pandemic virus [19]. In particular, Carlson *et al*. speculated that the protective role of TGF-β may involve modulation of immunopathology since TGF-β neutralization had a minimal effect on viral burden [19].

The mechanism responsible for TGF-β-mediated protection in asthmatic mice may involve prevention of tissue injury as opposed to augmented tissue repair. Newly identified cell types termed innate lymphoid cells (ILCs) participate in restoring airway epithelial integrity and lung tissue homeostasis during influenza virus infection through production of amphiregulin [44], which was in fact, reduced in asthmatic mice. Thus, it is likely that severe tissue injury did not occur in asthmatic mice that would have otherwise necessitated an ILC/amphiregulin response for tissue repair. This idea is further supported by the fact that neither BALF cytotoxicity nor total protein levels in asthmatic mice increased significantly above their respective pre-infection levels. However, an important question remains as to how TGF-β1 suppresses influenza virus-induced tissue damage. The most likely mechanism is that TGF-β1 preserves the overall integrity of the lung by directly inhibiting cytokine production from various immune cells. Indeed, asthmatic mice exhibited less severe asthma exacerbation following influenza infection. A homeostatic function for TGF-β in the lung has been previously described by Morris *et al*. [45]. In their work, the authors showed that active TGF-β in the lung can influence the pulmonary immune milieu by maintaining the anti-inflammatory state of alveolar macrophages through upregulation of expression of the inhibitory receptor, CD200 [46]. Consistent with the above reports, we also showed that in the absence of TGF-β receptor II, total protein levels increased, which indicated enhanced tissue injury, but without enhanced viral replication, and this correlated with loss of protection in asthmatic mice.

This study is the first to report an enhanced resistance of asthmatic mice to influenza that is dependent on TGF-β1. Ishikawa *et al*. have identified NK cells as the major mediator of increased resistance in asthmatic mice [47]. Their conclusions were based upon the use of anti-asialoGM1 serum to deplete NK cells and abrogate resistance. However, asialoGM1 is expressed on a wide variety of cells [48–53]. Indeed, a recent study showed that anti-asialoGM1 serum depletes, in addition to NK cells, basophils and, thereby abolishes IgE-mediated chronic cutaneous allergic inflammation [53]. Therefore, the reduced resistance in anti-asialoGM1 treated mice in the study by Ishikawa *et al*. might have been due to the simultaneous depletion of a number of different cell types and/or the lack of an asthmatic phenotype in the absence of basophils. In contrast, a specific depletion of NK cell using NK1.1 mAb did not reverse the resistance of asthmatic C57Bl/6 mice to influenza. This conclusively shows that the resistance to influenza observed in our model was not dependent on NK cells. Furthermore it is important to note that the asthma model used by Ishikawa *et al*. did not induce typical asthma phenotype. Thus, the apparent inconsistency between our observations and their results was not surprising.

The data presented in this study appear to contradict epidemiological findings suggesting that asthma is associated with increased rates of hospital admission during influenza, severe disease and death [54–59]. More recent reports, however, have suggested that among hospitalized patients during influenza pandemics, asthmatics are actually less likely to die compared to non-asthmatics [59,60]. Increased survival among hospitalized asthmatic patients during the 2009 pandemic may be due to the fact that these individuals are aware of their condition and seek medical care sooner than their non-asthmatic counterparts, and therefore, receive antiviral and/or corticosteroid treatment in a more timely fashion [61]. In our mouse model, asthmatic mice were not treated with corticosteroid or antiviral drugs and yet exhibited better survival following influenza infection. The apparent discrepancy between the human and mouse data may arise from differences in the timing of viral and allergic challenge. The sequence of events is critically important because an active form of TGF-β, a mediator of protection, is not constitutively expressed at high levels in asthmatic patients [62] or in mice. High expression of TGF-β above its baseline is inducible by an allergic antigen challenge but its expression is transient in nature as shown in this study. Given that only approximately 2% of all asthmatic patients have uncontrolled persistent allergic asthma [63] and that 85% of asthma exacerbations are triggered by viral infections [64], it can be speculated that most asthmatic patients do not have upregulated TGF-β expression at the time of viral challenge and therefore are not protected against viral infections. However, in our experiments, asthmatic mice were infected when TGF-β expression was highly upregulated by allergic challenge. Thus, primary infection of influenza-naïve adult mice after allergen induced airway inflammation may not necessarily accurately mimic the clinical setting and this may explain the discrepancy between human observational data and results from the present mouse study. It will be of considerable interest to directly determine whether non-viral induced asthma exacerbations in humans are followed by a transient period of increased resistance to influenza infection. To the best of our knowledge, such clinical data are not available.

We have recently reported that asthmatic mice are more susceptible to secondary heterologous challenge with CA04 virus [65]. In that study, we utilized a secondary influenza challenge model in which asthmatic mice were reinfected with influenza virus at week 6 post OVA challenge when TGF-β expression is at the baseline. We found that CA04-specific antibody responses were suppressed during secondary challenge and as a result, asthmatic mice were more susceptible to influenza infection. We now know that asthmatic mice express large amounts of active TGF-β for up to 5 weeks in the airway. Thus, it is likely that the lack of TGF-β responses at the time of secondary challenge failed to protect asthmatic mice and this further support the conclusion of the present study that asthma associated TGF-β response is protective but the protection is only transient. Based on our previous work and the present studies, we propose that future co-morbidity study of allergic airway inflammation and influenza should investigate the impact of asthma on the host susceptibility to viral infections when TGF-β is at the baseline, i.e when lung homeostasis has been restored after allergic challenge, to better mimic infections of asthmatic patients.

In conclusion, we report for the first time that increased expression of TGF-β1 in asthmatic mice confers resistance to influenza virus infection through suppression of tissue injury. Our study provides compelling evidence that TGF-β1 would have therapeutic potential in preventing influenza-related mortality. A therapeutic measure that is not dependent on antigen specificity is particularly important in the event of an influenza pandemic when antigen-matched vaccines are not immediately available. It will be of great interest to ascertain whether asthmatic mice are also protected against H5N1, a highly pathogenic strain known to cause an exacerbated cytokine storm.

## Materials and Methods

### Ethics statement

Animal care and experimental protocols were in accordance with the NIH “Guide for the Care and Use of the laboratory Animals” and were approved by the Institutional Animal Care and Use Committee at Albany Medical College (Protocol Number 11–04004).

### Co-morbidity mouse model of asthma and influenza

Eight-week-old C57BL/6 and BALB/c mice were purchased from Charles River Laboratories through a contract with the National Cancer Institute. BALB/c IFN-IR^-/-^ mice were kindly provided by Dr. Daniel Portnoy (University of California, Berkeley, CA) and mice were maintained under specific pathogen-free conditions in the Animal Research Facility at Albany Medical College. To induce acute allergic lung inflammation, the mice were first sensitized against OVA (Sigma-Aldrich) by injecting 10 μg of OVA in aluminum hydroxide (General Chemical) i.p. twice at weekly intervals. One week after the last immunization, the sensitized mice were anaesthetized with isoflurane and challenged i.n. with 100 μg of OVA in PBS for 5 consecutive days. Control, non-asthmatic mice were sensitized and challenged with PBS. At various time points after the final i.n. OVA inoculation, asthmatic and non-asthmatic mice were challenged i.n. with either CA04 or PR8. For HDM-asthma model, mice were anaesthetized with isoflurane and i.n. exposed to 50 μg of house dust mite (HDM) extract (*Dermatophagoides pteronyssinus*: Greer Laboratories) in PBS for three consecutive days every three weeks. Control non-asthmatic mice received PBS or LPS from *E*. *coli* 0111:B4 (0.64 endotoxin unit in 50uL PBS). At the indicated time points after the last HDM inoculation, asthmatic and non-asthmatic mice were infected i.n. with a 4LD_50_ of CA04. The influenza type A viruses were propagated in 10-day-old embryonated chicken eggs (Charles River) or in Madin-Darby canine kidney (MDCK) cells.

### Plaque assay

At indicated time points after i.n. challenge, bronchoalveolar lavage fluids (BALF) were harvested by lavaging the lungs with 1 ml of PBS. BALF were aliquoted and stored at -80°C for plaque assays. Serial dilutions of BALF were added to MDCK cells monolayers for viral plaque enumeration.

### Cytokine and granzyme B analysis

For cytokine analysis, BALF samples were centrifuged at 300 x *g* for 10 min at 4°C and the cell-free BALF samples were aliquoted and stored at -80°C. Protein levels of TGF-β1 in BALF were analyzed by either ELISA (eBioscience) or mouse Bio-Plex Luminex assays (Bio-Rad) following the manufacturers’ instructions. Levels of IL-5, IL-6, IL-10, IL-12, MCP-1, IFN-γ, and TNF in lung homogenates were measured by cytometric bead array (BD Biosciences). Granzyme B (gzmB) levels in BALF were determined by a mouse gzmB ELISA kit (eBioscience).

### Cytotoxicity assay

Cytotoxic levels were quantitated by Vybrant Cytotoxicity Assay Kit (Molecular Probes). In brief, this assay measures the cytosolic enzyme glucose 6-phosphagte dehydrogenase (G6PD) that is released from damaged cells into the BALF. G6PD generates NADPH, which in turn, leads to reduction of resazurin into red-fluorescent resorufin. The fluorescence signal was measured by a plate reader and the median fluorescence intensity (MFI) correlated with the number of dead cells.

### Total protein assay

The total protein levels in the cell free fraction of BALF were determined using a Bicinchoninic acid (BCA) protein assay kit (Thermo Scientific Pierce).

### Flow cytometry

The lungs were harvested and incubated with 0.25 mg/ml of DNAse I (Roche Diagnositics), 2 mg/ml of collagenase D (Roche Diagnostics), and 1 mM of MgCl_2_ for 45 min at 37°C. The digested lungs were passed through a 40 μm nylon cell strainer, followed by 5 min incubation with ammonium-chloride-potassium lysis buffer to obtain red blood cell-depleted single-cell suspensions. Live cells were enumerated based on trypan blue staining. 5 x 10^5^ cells were incubated with Fixable Viability Dye (FVD) (eFluor 780; eBioscience) for 30 min on ice, followed by incubation with 2.4G2 mAb (anti-mouse FcγIII/II receptor) for 15 min. Fc receptor-blocked cells were then stained with mixtures of anti-mouse surface antigen mAbs: anti-CD4 (clone GK1.5) (FITC; BD Pharmingen), anti-CD19 (clone 1D3) (PE; BD Pharmingen), anti-CD8 (clone 53–6.7) (PE-Cy7; BD Pharmingen), anti-F4/80 (clone BM8) (PE; eBioscience), anti-CD11c (clone N418) (APC-Cy7; BioLegend), anti-Ly6G (clone 1A8) (PE-Cy7; Biolegend), anti-CD11b (clone M1/70) (FITC; eBioscience), anti-CD326/EpCAM (clone 48.8) (PE; eBioscience), anti-CD3 (clone 145-2C11) (FITC; BD Pharmingen), anti-CD45 (clone 30-F11) (PE-Cy7; BioLegend), anti-Siglec F (clone E50-2440) (PE; BD Pharmingen), anti-CD49b (clone Dx5) (FITC; eBioscience), and anti-LAP (clone TW7-16B4) (APC; BioLegend). Stained cells were analyzed using a FACSCanto flow cytometer. The cell debris was gated out based on forward and side scatter and live cells were gated in based on FVD staining. Median fluorescence intensity (MFI) of APC–conjugated anti-mouse LAP was measured for the indicated subsets of cells. To analyze influenza A virus nucleoprotein (NP)-specific CD8^+^ T cells, cells were further stained with APC-conjugated H-2K^d^ NP tetramer (TYQRTRALV) for 45 min at 4°C. The tetramer was obtained through the NIH Tetramer Facility.

### TβRII conditional knockout mice

Floxed *TβRII* (*TβRII* ^*f/f*^) and *Ubc-CreER* ^*T2*^ (*Cre*) mice were purchased from The Jackson Laboratory. *TβRII* ^*f/f*^ mice were crossed with tamoxifen inducible *Cre* transgenic mice to generate homozygous *TβRII* conditional knockout (*TβRII* ^*f/f*^ *-Cre)* mice, heterozygous *TβRII* knockout mice (*TβRII* ^*f/+*^ *-Cre)* and control mice (*TβRII* ^*f/f*^, *TβRII* ^*f/+*^, *TβRII* ^*+/+*^ *-Cre)*. For the induction of Cre, 2 mg of tamoxifen (Sigma-Aldrich) in corn oil (Sigma-Aldrich) was injected i.p. once/day for 5 consecutive days. OVA-sensitized mice were treated with tamoxifen prior to i.n. OVA treatment. Cre recombinase-mediated deletion of the floxed *TβRII* gene was confirmed by PCR for *TβRII* ^*null*^ and flow cytometry analysis for TGF-βRII expression (S8 Fig).

### Influenza A virus-specific antibodies

Cell-free BALF samples collected on day 7 post-CA04 virus infection were analyzed for antigen-specific antibodies by ELISA. A 96-well MaxiSorp plate (Nunc) was coated with 2 μg/ml of H1N1 A/California/09/2009 monovalent vaccine (Sanofi Pasteur) and incubated at 4°C overnight. Wells were then washed with PBS + 0.05% Tween (Sigma) and blocked with PBS + 1% FCS for 2 hr at room temp. After washing, the wells were incubated with serial 2-fold sample dilutions for 2 hr at room temp. The plates were then incubated with biotin-conjugated goat anti-mouse antibodies specific for IgG1, IgG2A, or IgG2b (Caltag Laboratories, Burlingame, CA) for 1 hr and then horseradish peroxidase-conjugated streptavidin was added (Biosource). After 30 min incubation, the plates were extensively washed and TMB peroxidase substrate (BD Biosciences) was added. The reaction was stopped by adding 1.8N H_2_SO_4_ and optical density was measured at 450 nm using a Power-Wave HT microplate reader (BioTek Instruments). Antibody titer is expressed as the reciprocal dilution that gave 50% of the maximum optical density.

### Hemagglutination inhibition (HI) assay

Serially-diluted cell free-BALF was mixed with 4 hemagglutination units of CA04 virus in V-bottom 96-well plates. After 30 min incubation, 0.5% of chicken red blood cells (Lampire Biological Laboratories) were added and incubated for additional 1 hr at room temp. The HI titer was defined as the reciprocal of the last dilution that prevented viral hemagglutination activity.

### In vivo immune cell depletion prior to CA04 virus challenge

Various immune cells including neutrophils, phagocytic cells, NK cells and T cells were depleted *in vivo* after the last OVA/PBS inoculation but before CA04 virus infection. For T cell depletion, mice were treated i.p. with 0.5 mg of anti-CD4 (clone GK1.5) (Bio X Cell) and anti-CD8 (clone 53–6.72) (Bio X Cell) mAbs on days 1, 2, 3, 4, and 5, and challenged with CA04 virus on day 7. For neutrophil depletion, mice were injected i.p. with 0.5 mg of anti-Ly-6C/G (RB6-8C5) (Maine Biotechnology) mAb on days 4 and 5. For phagocyte depletion, 50 ul of liposomes containing clodronate (clodronateliposomes.com) were administered i.n. on days 4 and 5. For NK cell depletion, 0.5 mg of NK1.1 (PK-136) (Maine Biotechnology) mAb was injected i.p. on days 4 and 5. Rat IgG and liposomal-PBS were used as controls for the above depletion experiments. The efficiency of cell depletion in the lungs (ranging from 80 to 99%) was confirmed one day prior to infection (day 6) by flow cytometry.

### Statistical analysis

Data were analyzed by unpaired Student’s *t* test with Welch correction for comparison of two groups and one- or two-way ANOVA with Bonferroni correction for comparison of multiple groups. Survival data were analyzed with log-rank (Mantel-Cox) test using GraphPad Prism 6 software (San Diego, CA). A *P* value of < 0.05 was considered to be statistically significant.

## Supporting Information

S1 Text Supporting Methods. (DOCX)

S1 Fig A mouse model of OVA-induced asthma. (**A**) Lungs were harvested at days 1 and 7 post-OVA treatment for histological analysis (4 mice/group). Hematoxylin and eosin stained lung sections were scored for levels of peribronchial inflammation as described in Methods and Materials. (**B** to **D**) Invasive lung function measurements with a mechanical ventilator were performed on days 1 and 7 post-OVA treatment. Newtonian resistance (*R*_*N*_) (**B**), tissue damping (*G*) (**C**), and tissue elastance (*H*) (**D**) were assessed in response to methacholine challenge (3–4 mice/group). **P<0.01, ***P<0.001.
(TIF)

S2 Fig Kinetics of Th-2 cytokine expression following acute asthma. Mice were treated with OVA as shown in Fig 1A. Lungs were harvested at days 1, 7, and 28 post-OVA treatment for cytokine analysis. Cytokine protein levels were measured by either cytometric bead array or ELISA. Each bar represents mean ± SD of 3–4 mice/group. **P<0.01; ****P<0.0001.
(TIF)

S3 Fig A mouse model of HDM-induced asthma. **(A)** A mouse model of HDM-induced asthma. Mice received were inoculated i.n. with HDM three times/week for three weeks. (**B** to **D**) Control, non-asthmatic mice received PBS only. On day 3 after the last HDM challenge, airway cellular infiltration (**B**) (5 mice/group), total serum IgE levels (**C**) 4–5 mice/group), and pulmonary functions were assessed (**D**) (3 mice/group). HDM-induced asthmatic and non-asthmatic mice were i.n. challenged with CA04 virus either 1, 2, or 3 weeks following HDM challenge. (**E**) Mice were monitored for weight loss for 20 days (5 mice/group). *P<0.05; ***P<0.001.
(TIF)

S4 Fig OVA sensitization and challenge is required for the increased survival of asthmatic mice. Asthmatic mice were sensitized and challenged with OVA. Non-asthmatic mice were either sensitized or challenged with OVA. Additonal control mice were treated i.n with *E*. *coli* LPS (0.63 endotoxin unit). Mice were infected with CA04 virus and monitored for survival (5–8 mice per group). *P<0.05
(TIF)

S5 Fig CD4^+^ T cells and Th17 cells are not required for resistance of asthmatic mice. (A) Absolute cell numbers of Tregs were identified with FoxP3 (clone FJK-16s) and CD4 (clone RM4.4) mAbs (5 mice per group). (**B** to **C**) CD4^+^ T cells were depleted by i.p. injection of GK1.5 mAb. Control mice were treated with non-specific rat IgG. Successful CD4^+^ T cell depletion in the lungs was confirmed by flow cytometry using fluorescent conjugated anti-CD4 (clone RM4.4) and anti-CD3 (clone 145-2C11) mAbs. Representative dot plots are shown (**B**). Control and CD4^+^ T cell depleted non-asthmatic and asthmatic mice were infected with CA04 virus and monitored for survival (7–8 mice per group) (**C**). (D) The concentrations of IL-17 were measured by Luminex (5 mice per group). (**E**) Non-asthmatic and asthmatic IL-17R^-/-^ mice were challenged with CA04 virus and were monitored for survival (7–8 mice/group). *P<0.05, ***P<0.001.
(TIF)

S6 Fig Early innate immune responses in asthmatic mice following CA04 infection. (**A**) IFN-α levels in BALF during influenza infection were measured by ELISA (3–5 mice/group). (**B** to **D**) Single-cell suspensions derived from mouse lungs were stained for neutrophils (**b**), macrophages (**C**), and NK cells (**D**). Each bar represents mean ± SD of 4 mice/group. **P<0.01; ***P<0.001; ****P<0.0001.
(TIF)

S7 Fig Asthmatic mice exhibit a reduced cytokine storm following PR8 challenge. Mice were treated with OVA as shown in Fig 1A. Asthmatic and non-asthmatic mice were infected with 4LD_50_ (2000 PFU) of PR8 virus and BALF were harvested on day 7 post-infection for cytokine analysis. Cytokine protein levels were measured by Bio-Plex cytokine assay. Each bar represents mean ±SD of 3–4 mice/group. *P<0.05.
(TIF)

S8 Fig Reduced ILC2 cytokine expression in asthmatic mice. (**A** to **C**) ILC2 cytokines were measured in either lung homogenate supernatants or BALF at various times after influenza infection. Amphiregulin (**A**) and IL-13 (**B**) levels were measured by ELISA and IL-5 (**C**) levels were quantified using cytometric bead array assay. Each bar represents mean ± SD of 3–5 mice/group. **P<0.01, **P<0.01, ****P<0.0001.
(TIF)

S9 Fig Influenza virus triggers reduced airway hyperresponsiveness in asthmatic mice. (**A**) The general experimental procedure used in this study. Invasive lung function measurements with a mechanical ventilator were performed on day 7 post influenza infection or PBS treatment (equivalent to day 14 post OVA challenge). (**B**) Newtonian resistance (*R*_*N*_), tissue damping (*G*), and tissue elastance (*H*) were assessed in response to methacholine challenge (4 mice/group). ***P<0.001.
(TIF)

S10 Fig OVA-induced asthma has minimal impact on sialic acid receptor expression. (**A** and **B**) Lungs were harvested on day 7 post-OVA challenge and single cell suspensions were prepared for flow cytometry analysis. Expression of α-2, 3 and α-2, 7 sialic acid linked receptors was measured on CD3^-^CD45^-^CD326^+^ epithelial cells. Representative flow cytometry histograms (**A**) and median fluorescent intensities (**B**) are shown. Each bar represents mean MFI ± SD (4 mice/group). *P<0.05.
(TIF)

S11 Fig Tamoxifen treatment of *TβRII* ^*f/f*^ *-Cre* mice leads to reduced TGF-βRII expression and deletion of floxed *TβRII* allele. (**A** and **B**) The number of TGF-βRII^+^ lung cells (**A**) and splenocytes (**B**) after 5 days i.p. treatment with corn oil or tamoxifen. (**C**) Cell surface expression of TGF-βRII was measured 2 days before CA04 challenge. (**D**) PCR screening for mice containing the *TβRII* null allele (2 mice/group). Each bar represents mean ± SD of 2 mice/group. *P<0.05. Data are representative of two independent experiments.
(TIF)
