# Supplementary material for: Prevention of Influenza Virus-Induced Immunopathology by TGF-β Produced during Allergic Asthma
Source: PLoS Pathog. 2015 Sep 25;11(9):e1005180. doi: 10.1371/journal.ppat.1005180 (PMC4583434; doi:10.1371/journal.ppat.1005180)
Supplement: S1 Text — (DOCX) [file ppat.1005180.s001.docx]

**Supplementary Information**

**Materials and Methods**

Airway hyperresponsiveness

Anaesthetized mice were tracheostomized and connected to a computer controlled small animal ventilator (flexiVent; SCIREQ). Mice were then paralyzed by i.p. injection with pancuronium bromide. Increasing doses of methacholine (0 – 30 mg/ml) were administered by nebulizer to monitor airway hyperresponsiveness.

Histology

Formalin-fixed lung sections were stained with hematoxylin and eosin (H&E). Slides were blinded and sections were scored for inflammatory infiltration as previously described [[66](#_ENREF_66)]. Peribronchial regions were scored for severity (0, normal; 1, <3 cell diameter thick; 2, 3-10 cells thick; 3, >10 cells thick) and extent (0, normal; 1, <10% of sample; 2, 10-25% of sample; 3, >25% of sample). Scores were calculated by multiplying severity by extent.

Total IgE antibodies

To measure IgE, 96 maxisorp plates (Corning, NY) were coated with 2 µg/ml of goat anti-mouse IgE (BD Pharmingen) diluted in PBS (Life Technologies) and incubated overnight at 4 ℃. Diluted samples and purified mouse IgEκ standards (BD Biosciences) were added to the plates, followed by washing and blocking with 2.5% FBS/PBS. Following a 1 hr incubation, the plates were washed and biotinylated goat anti-mouse IgE-biotin (Caltag, Burlingame, CA) diluted to 0.5 µg/ml was added. After washing, streptavidin-horseradish peroxidase (Biosource, Camarillo, CA) was added and the plates were incubated for 30 min. After incubation, the plates were washed and developed with TMB peroxidase substrate (KPL, Gaithersburg, MD). Color development was stopped with 1.8N sulfuric acid and the plates were read at 450 nm using a PowerWave HT microplate reader (BioTek Instruments, Winooski, VT).

Determination of endotoxin contamination levels

OVA (14.5 endotoxin unit/ml) and HDM (12.8 endotoxin unit/ml) stocks were assayed for endotoxin levels using Pierce LAL Chromogenic Endotoxin Quantification Kit (Thermo Scientific).

Regulatory T analysis

For intracellular staining, CD4^+^ T cells were fixed, permeabilized (Cytofix/Cytoperm kit; BD Bioscience), and stained with FITC-conjugated anti-FoxP3 (clone FJK-16s; BD Biosciences). Stained samples were acquired on FACS Canto (BD Biosciences).

Confirmation of in vivo CD4^+^ T cell depletion

*In vivo* depletion of CD4^+^ T cells were confirmed by flow cytometry analysis. Red blood cell-depleted, lung single cell suspensions were first incubated with Fixable Viability Dye (eFluor 780; eBioscience), followed by incubation with anti-mouse FcγRIII/II receptor (2.4G2) mAb. Cells were then stained with a cocktail of anti-mouse CD4 (clone RM4-5) (APC; BD Pharmingen) and anti-mouse CD3 (clone 145-2C11) (FITC; BD Pharmingen). Stained cells were analyzed using a FACSCanto.

Flow cytometric analysis of sialic acid and TGF-βRII receptors

For analysis of sialic acid receptor expression on epithelial cells, lung cells were incubated with biotinylated Maackiaamurensis lectin II (MAAII) or Sambucusnigra agglutinin (SNA)(Vector Laboratories) which are specific for alpha-2,3 (α-2,3 SA) and alpha-2,6 (α-2,6 SA) sialic acid-linked receptor, respectively, and bound lectin was detected using streptavidin (Alexafluor 488)(Invitrogen). The MFI of positively-stained CD3^-^CD45^-^EpCAM^+^ epithelial cells was determined. For TGF-βRII expression, cells were stained with APC-conjugated anti-TGF-βRII polyclonal Ab (R&D Systems). The MFI of stained cells was determined using a FACSCanto flow cytometer.

Cytokine analysis

For cytokine analysis, lung homogenates or BALF samples were centrifuged at 300 x *g* for 10 min at 4^o^C and the cell free samples were aliquoted and stored at -80^o^C. Cytokine levels were analyzed using ELISA kits from eBioscience (IL-13) and R&D Systems (amphiregulin). Protein levels of IL-5 in lung homogenate samples were measured by cytometric bead array (BD Biosciences). Concentrations of IL-17 were measured with the Milliplex MAP Mouse Cytokine kit (Millipore). For IFN-α ELISA, monoclonal rat anti-mouse IFN-α (HyCult Biotechnology) was used as a capture antibody and polyclonal rabbit anti-mouse IFN-α (PBL Interferon Source) for detection of bound IFN-α. Recombinant mouse IFN-α (HyCult Biotechnology) was used to establish a standard curve.

Genotyping of TβRII conditional knockout mice

Cre recombinase-mediated deletion of the floxed *TβRII* gene was confirmed by PCR for *TβRII^null^* allele as previously described [[67](#_ENREF_67)] using the primers: 5’-TATGGACTGGCTGCTTTTGTATTC-3′ and 5′-TATTGGGTGTGGTTGTGGACTTTA-3′ that result in a 692 bp product for null allele and no product for non Cre-recombinant mice.
