# Supplementary figures and images for: Prevention of Influenza Virus-Induced Immunopathology by TGF-β Produced during Allergic Asthma
Source: PLoS Pathog. 2015 Sep 25;11(9):e1005180. doi: 10.1371/journal.ppat.1005180 (PMC4583434; doi:10.1371/journal.ppat.1005180)

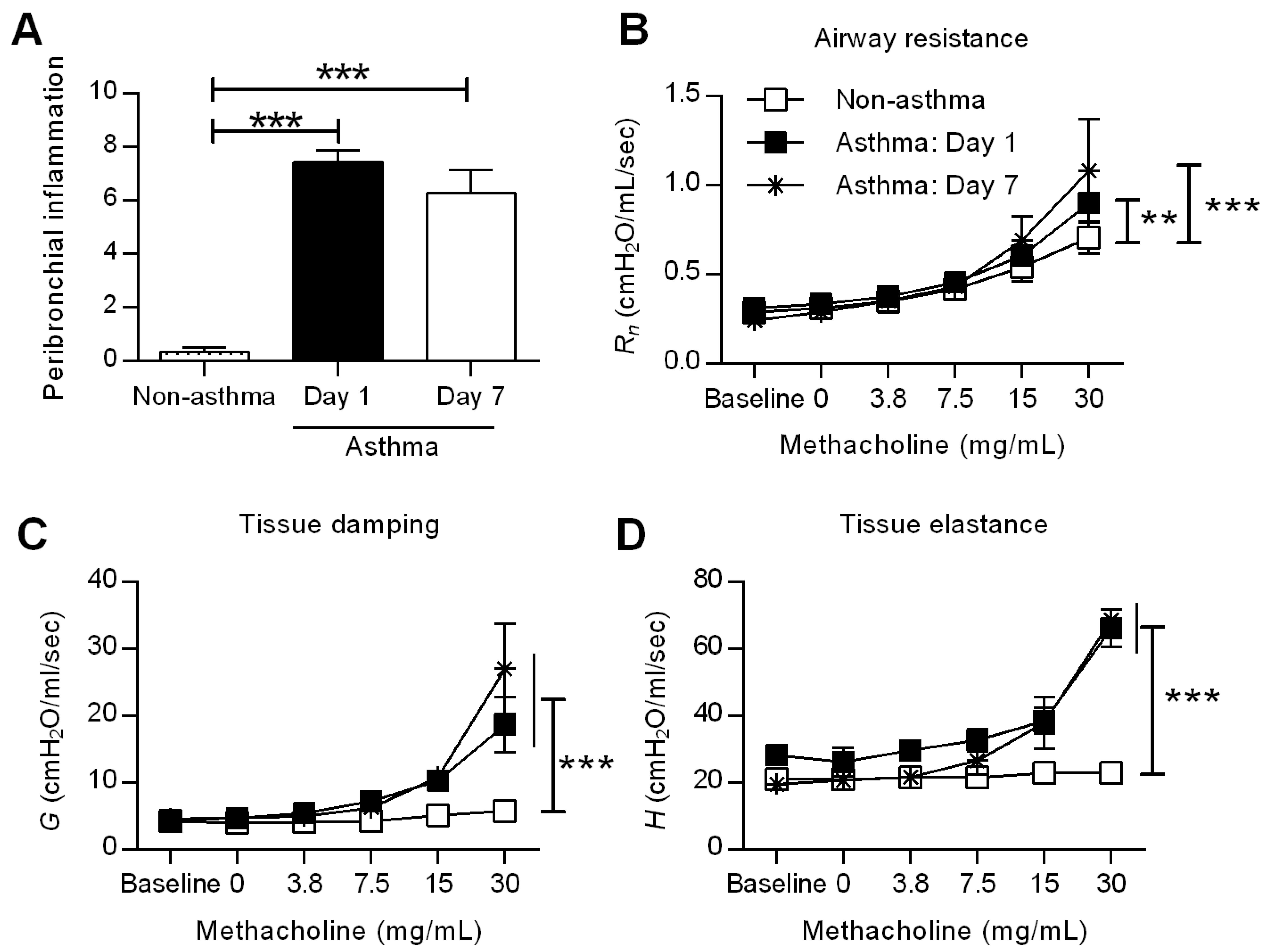

Supplement: S1 Fig — (A) Lungs were harvested at days 1 and 7 post-OVA treatment for histological analysis (4 mice/group). Hematoxylin and eosin stained lung sections were scored for levels of peribronchial inflammation as described in Methods and Materials. (B to D) Invasive lung function measurements with a mechanical ventilator were performed on days 1 and 7 post-OVA treatment. Newtonian resistance (R N) (B), tissue damping (G) (C), and tissue elastance (H) (D) were assessed in response to methacholine challenge (3–4 mice/group). **P<0.01, ***P<0.001. (TIF) [file ppat.1005180.s002.tif]

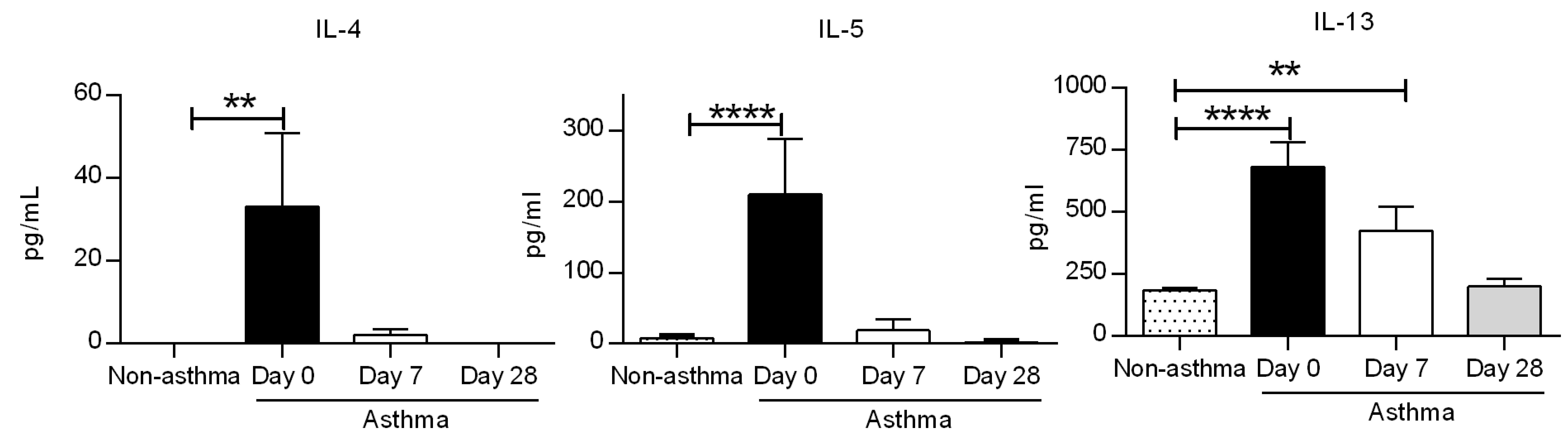

Supplement: S2 Fig — Mice were treated with OVA as shown in Fig 1A. Lungs were harvested at days 1, 7, and 28 post-OVA treatment for cytokine analysis. Cytokine protein levels were measured by either cytometric bead array or ELISA. Each bar represents mean ± SD of 3–4 mice/group. **P<0.01; ****P<0.0001. (TIF) [file ppat.1005180.s003.tif]

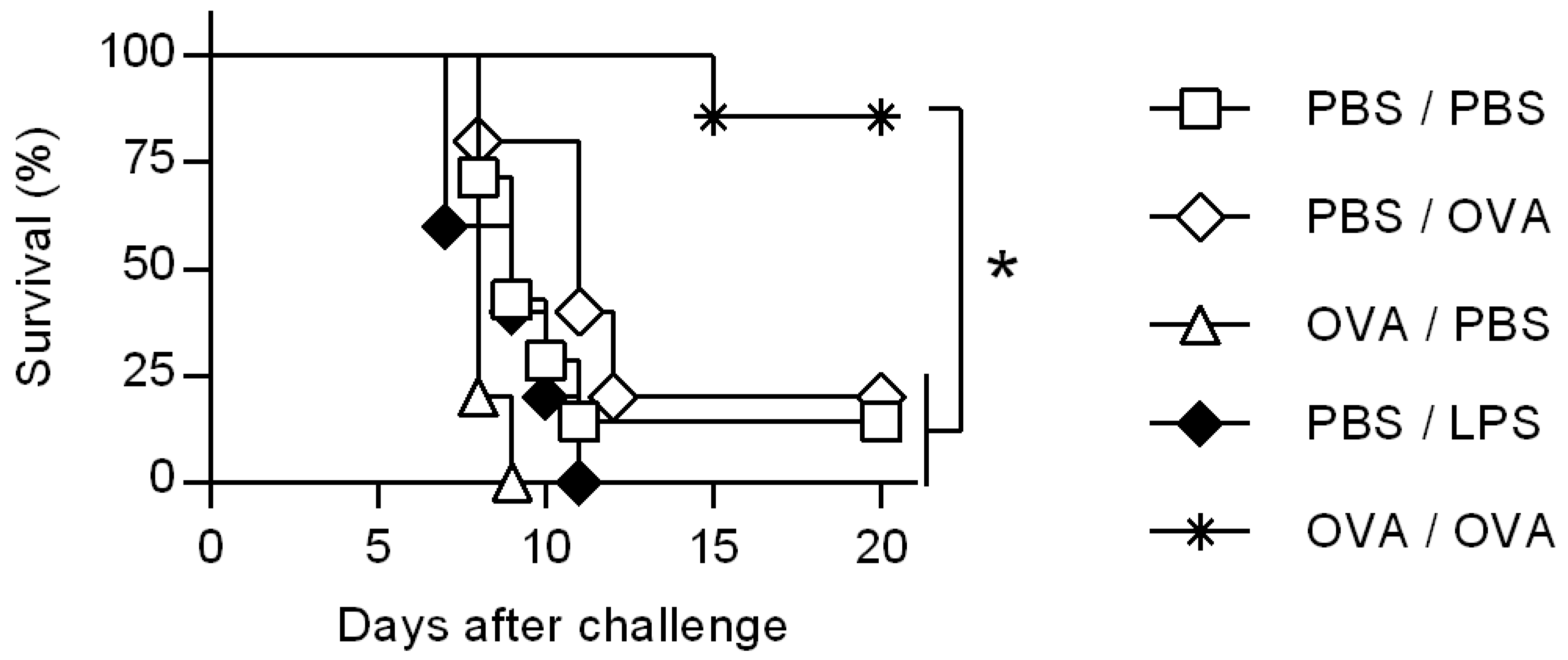

Supplement: S4 Fig — Asthmatic mice were sensitized and challenged with OVA. Non-asthmatic mice were either sensitized or challenged with OVA. Additonal control mice were treated i.n with E. coli LPS (0.63 endotoxin unit). Mice were infected with CA04 virus and monitored for survival (5–8 mice per group). *P<0.05 (TIF) [file ppat.1005180.s005.tif]

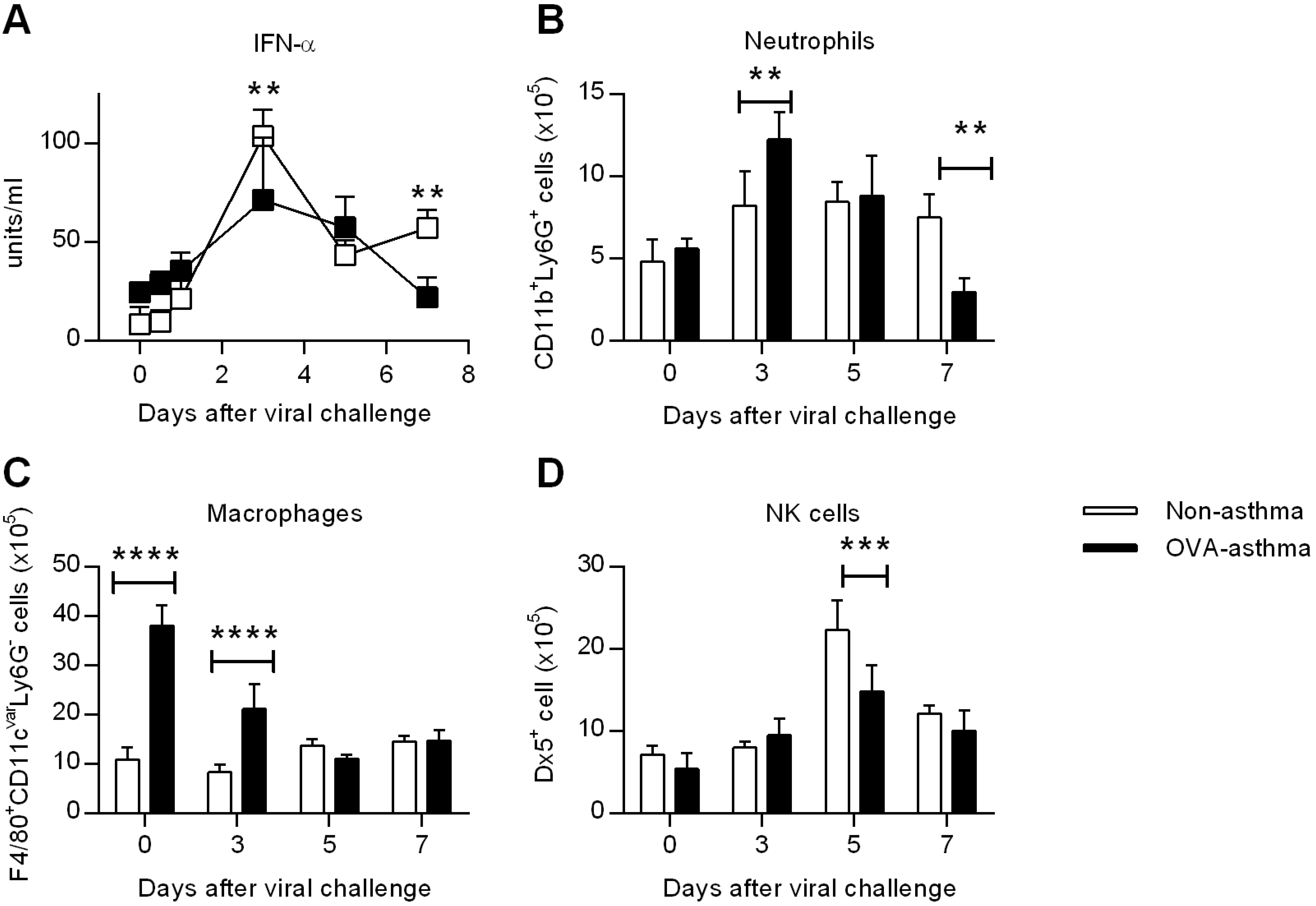

Supplement: S6 Fig — (A) IFN-α levels in BALF during influenza infection were measured by ELISA (3–5 mice/group). (B to D) Single-cell suspensions derived from mouse lungs were stained for neutrophils (b), macrophages (C), and NK cells (D). Each bar represents mean ± SD of 4 mice/group. **P<0.01; ***P<0.001; ****P<0.0001. (TIF) [file ppat.1005180.s007.tif]

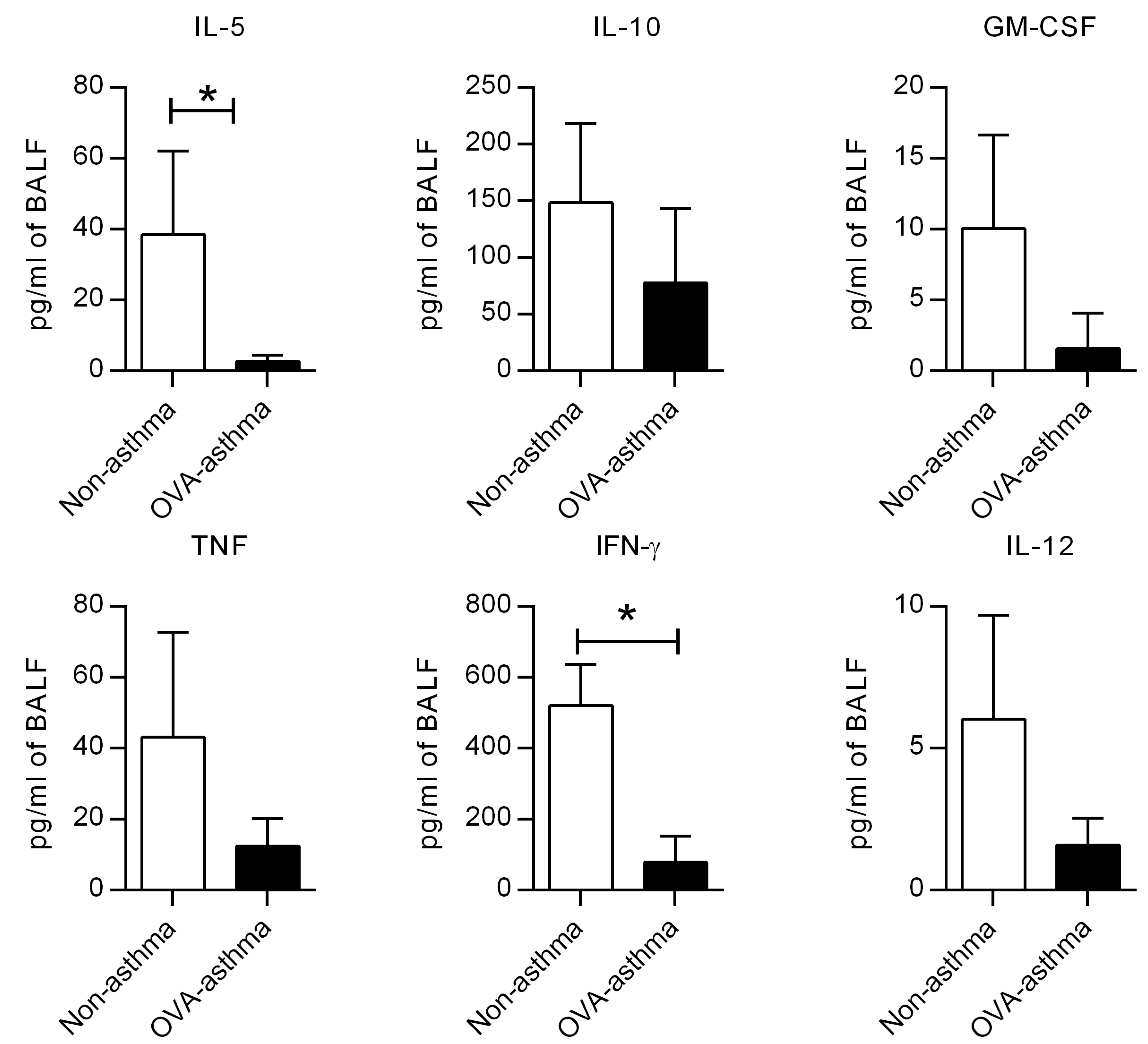

Supplement: S7 Fig — Mice were treated with OVA as shown in Fig 1A. Asthmatic and non-asthmatic mice were infected with 4LD50 (2000 PFU) of PR8 virus and BALF were harvested on day 7 post-infection for cytokine analysis. Cytokine protein levels were measured by Bio-Plex cytokine assay. Each bar represents mean ±SD of 3–4 mice/group. *P<0.05. (TIF) [file ppat.1005180.s008.tif]

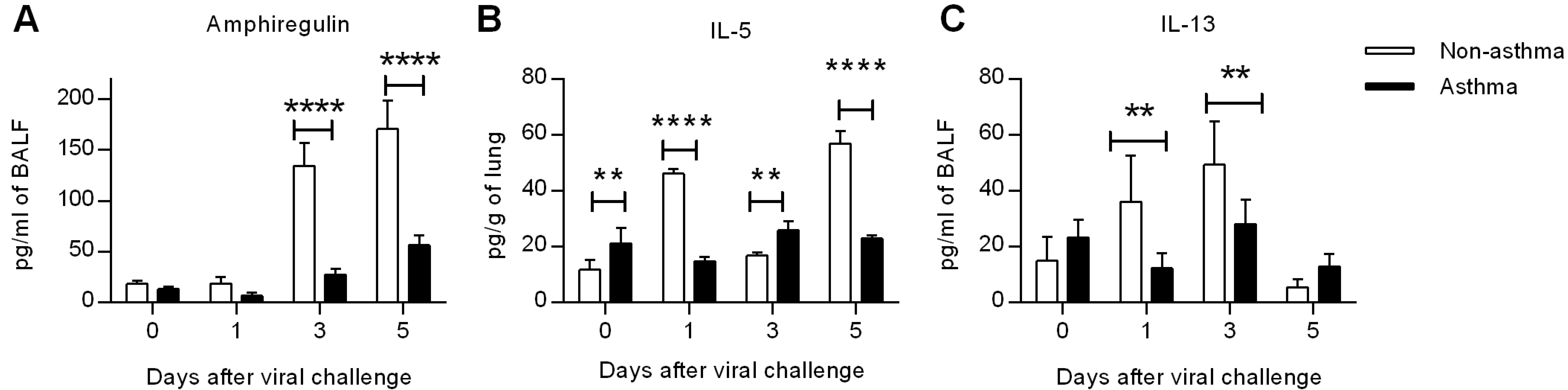

Supplement: S8 Fig — (A to C) ILC2 cytokines were measured in either lung homogenate supernatants or BALF at various times after influenza infection. Amphiregulin (A) and IL-13 (B) levels were measured by ELISA and IL-5 (C) levels were quantified using cytometric bead array assay. Each bar represents mean ± SD of 3–5 mice/group. **P<0.01, **P<0.01, ****P<0.0001. (TIF) [file ppat.1005180.s009.tif]

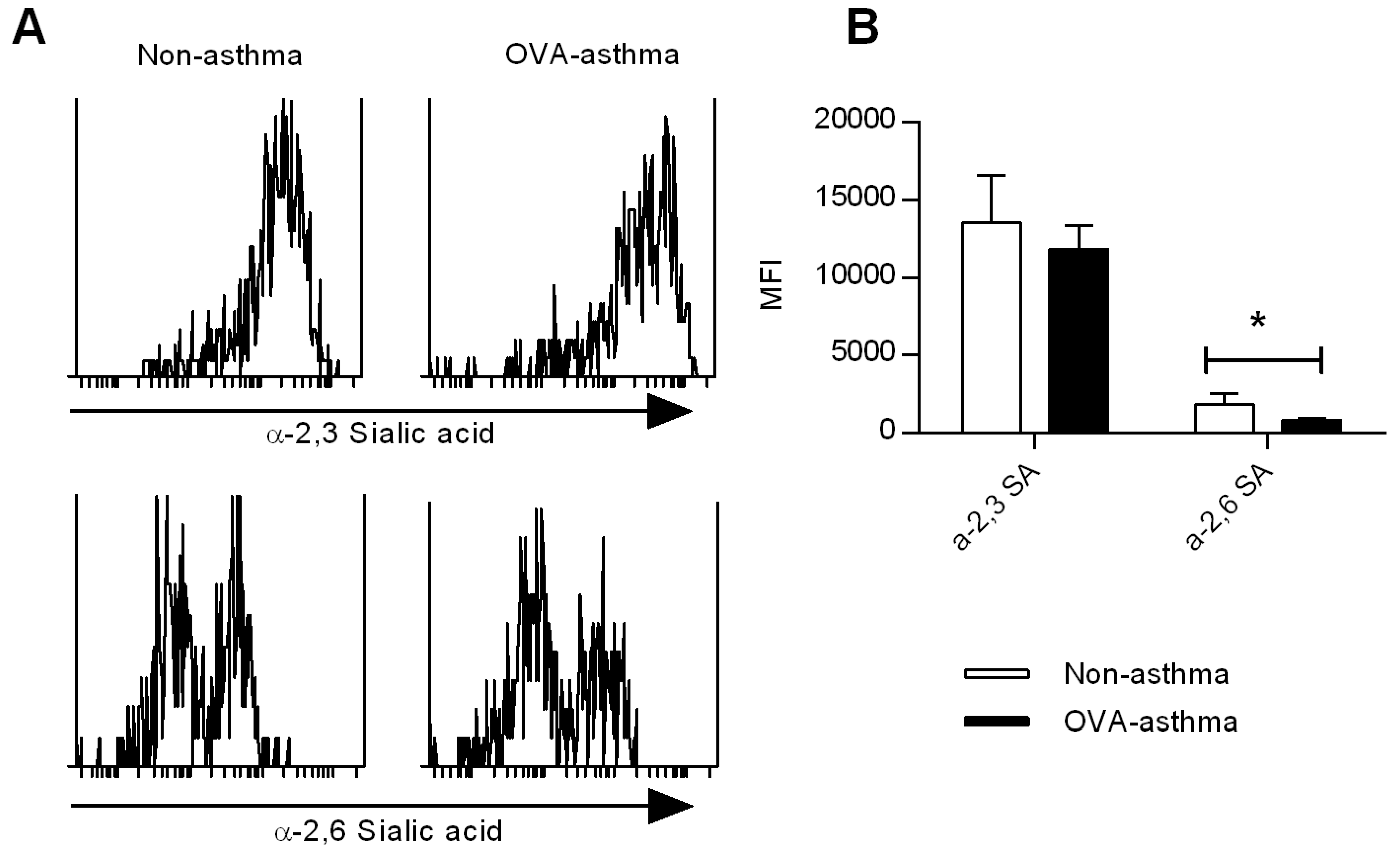

Supplement: S10 Fig — (A and B) Lungs were harvested on day 7 post-OVA challenge and single cell suspensions were prepared for flow cytometry analysis. Expression of α-2, 3 and α-2, 7 sialic acid linked receptors was measured on CD3-CD45-CD326+ epithelial cells. Representative flow cytometry histograms (A) and median fluorescent intensities (B) are shown. Each bar represents mean MFI ± SD (4 mice/group). *P<0.05. (TIF) [file ppat.1005180.s011.tif]

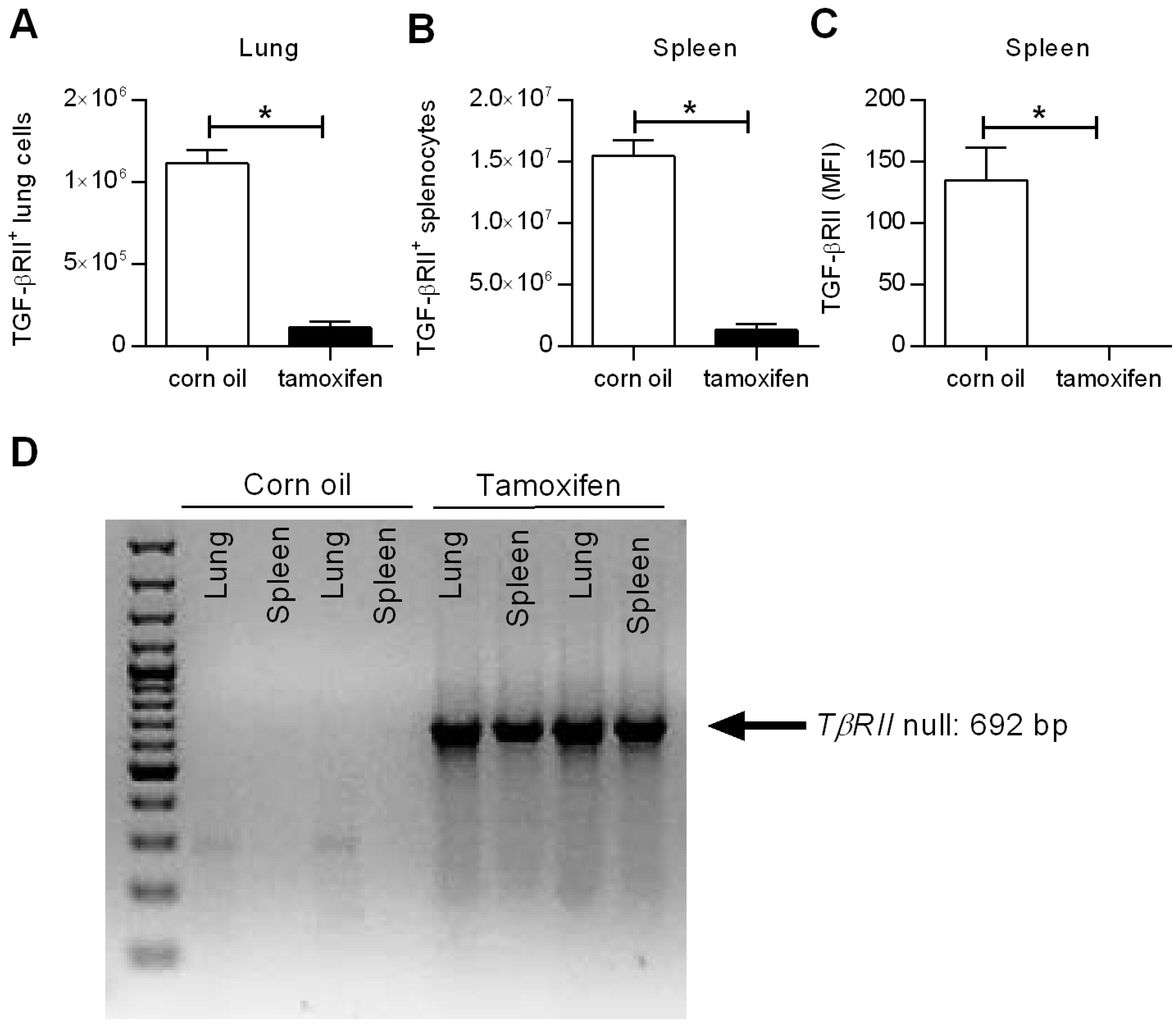

Supplement: S11 Fig — (A and B) The number of TGF-βRII+ lung cells (A) and splenocytes (B) after 5 days i.p. treatment with corn oil or tamoxifen. (C) Cell surface expression of TGF-βRII was measured 2 days before CA04 challenge. (D) PCR screening for mice containing the TβRII null allele (2 mice/group). Each bar represents mean ± SD of 2 mice/group. *P<0.05. Data are representative of two independent experiments. (TIF) [file ppat.1005180.s012.tif]
